# Towards an emerging role for anticoagulants in cancer therapy: a systematic review and meta-analysis

**DOI:** 10.3389/froh.2024.1495942

**Published:** 2024-11-06

**Authors:** Huda Moutaz Asmael Al-Azzawi, Syed Ameer Hamza, Rita Paolini, Fizza Arshad, Romeo Patini, Lorraine O’Reilly, Michael McCullough, Antonio Celentano

**Affiliations:** ^1^ Melbourne Dental School, The University of Melbourne, Carlton, VIC, Australia; ^2^ Head and Neck Department, “Fondazione Policlinico Universitario A. Gemelli—IRCCS” School of Dentistry, Catholic University of Sacred Heart—Rome Largo A. Gemelli, Rome, Italy; ^3^ Clinical Translation Centre, Cancer Biology and Stem Cells Division and Inflammation Division, The Walter and Eliza Hall Institute of Medical Research, Parkville, VIC, Australia; ^4^ Department of Medical Biology, University of Melbourne, Parkville, VIC, Australia

**Keywords:** cancer, preclinical mice models, anticoagulants, heparin, warfarin, NOACs

## Abstract

**Background:**

Anticoagulants, renowned for their role in preventing blood clot formation, have captivated researchers’ attention for the exploitation of their potential to inhibit cancer in pre-clinical models.

**Objectives:**

To undertake a systematic review and meta-analysis of the effects of anticoagulants in murine cancer research models. Further, to present a reference tool for anticoagulant therapeutic modalities relating to future animal pre-clinical models of cancer and their translation into the clinic.

**Methods:**

Four databases were utilized including Medline (Ovid), Embase (Ovid), Web of science, and Scopus databases. We included studies relating to any cancer conducted in murine models that assessed the effect of traditional anticoagulants (heparin and its derivatives and warfarin) and newer oral anticoagulants on cancer.

**Results:**

A total of 6,158 articles were identified in an initial multi-database search. A total of 157 records were finally included for data extraction. Studies on heparin species and warfarin demonstrated statistically significant results in favour of tumour growth and metastasis inhibition.

**Conclusion:**

Our findings constitute a valuable reference guide for the application of anticoagulants in cancer research and explore the promising utilization of non-anticoagulants heparin in preclinical cancer research.

**Systematic Review Registration:**

PROSPERO [CRD42024555603].

## 1 Introduction

Despite advances in surgery, imaging technologies, and new targeted treatment modalities for advanced cancers, cancer remains the leading cause of death globally, accounting for approximately 10 million deaths in 2020 (1). In 2020, it was estimated that 10 million lives were lost due to cancer and it is suspected that, by 2040, around 28 million people will have been newly diagnosed with cancer (2).

Cancer is an umbrella term that encompasses a large group of diseases involving any part of human body (3). Several risk factors have been documented as contributing to cancer initiation, including tobacco smoking, alcohol, ionizing radiation, electromagnetic field, UV light, dietary factors, lack of physical activity, infections, and chemical exposure (4). Cancer therapy depends on the type and location of the cancer and degree of invasiveness. Currently, multiple therapeutic options are available for cancer as primary and/or secondary adjuvant therapies, that include but not limited to; surgery, chemotherapy, radiotherapy, immunotherapy, CAR T cell therapy, hormone therapy, anti-angiogenic treatment, stem cell therapy, ablation therapy, targeted therapy, photodynamic therapy, sonodynamic therapy, chemodynamic therapy, ferroptosis-based therapy, and cancer mRNA vaccines in development (5, 6). Murine preclinical models are important tools in oncology research due to their 90% genetic similarity to humans enabling researchers to study cancer biology (7), test new therapy combinations, and expedite the development of novel treatments to enhance patient outcomes (7).

Cancer patients are at a higher risk of developing venous thromboembolism (VTE), (approximately 4–7 times) compared to non-cancer patients, with 15% experiencing venous thromboembolic events (8). Therefore, anticoagulants (ACs) are commonly prescribed for these patients to prevent cancer-associated thrombosis (9). Furthermore, a significant number of cancer patients may already be on concurrent AC treatment for systemic reasons. As an example, in our recently conducted multicentre study across three Australian hospitals, we found out that approximately 6.5% of oral cancer patients were being treated simultaneously with ACs for systemic purposes (unpublished data).

The debate over AC efficacy as anti-cancer agents has been ongoing for over 50 years. However, there is now considerable evidence derived from both *in vitro* and *in vivo* studies to indicate that conventional ACs (heparin and warfarin) are not only effective for blood clot formation prevention, but also may exert anti-cancer effects (10, 11). Heparin and its derivatives have been shown to have anti-metastatic properties in multiple preclinical animal's studies, including impeding cancer cell proliferation, adhesion, invasion and metastasis. These have been shown to occur through multiple mechanisms, such inhibition of heparanase, P-L selectin mediated-cell adhesion, angiogenesis, and inhibition of lymphogenesis process via the VEGF-C/VEGFR-3 axis. However, ACs have limitations that are dose dependent, including excessive bleeding and heparin induced thrombocytopenia (HIT) (10–12).

Historically, warfarin was the oral AC drug of choice for more than half century until the recent advent of novel oral anticoagulants drugs (NOAC). Notably, warfarin has a narrow therapeutic window due to its broad drug and food interactions (13, 14). However, the mechanism behind the proposed anti-cancer properties appears not to be related to its anticoagulation properties, rather due to inhibition of the receptor tyrosine kinase Axl that is associated with cancer cell proliferation, migration and invasiveness (15). NOACs are novel classes of anticoagulants, including the direct thrombin inhibitor (dabigatran) and factor Xa inhibitor (edoxaban, rivaroxaban and apixaban) (16). The National Comprehensive Cancer Network (NCCN) and the International Society on Thrombosis and Haemostasis (ISTH) recommended the use of NOAC as an alternative to low molecular weight heparin (LMWH) and warfarin for the treatment of cancer associated thrombosis (16).

We designed our systematic review and meta-analysis to address the following key questions using the PICOS framework: In preclinical murine models of cancer (P), how does the administration of traditional anticoagulants (such as heparin and warfarin) and newer oral anticoagulants (I), compared to placebo or no treatment (C), affect cancer biology? Additionally, what are the most common routes of administration, dosage protocols, and therapeutic time windows for anticoagulant administration concerning tumour initiation and progression (O) in *in vivo* preclinical studies (S)?

To the best of the authors’ knowledge, this is the first study investigating and achieving the above aims. We comprehensively summarise the knowledge base regarding the effects of both traditional ACs (heparin and its derivatives and warfarin) and novel oral ACs (NOACs) in relation to cancer therapy in preclinical murine studies, which may have clinical translative ramifications.

## 2 Methods

### 2.1 Study design

This systematic review and meta-analysis was conducted in accordance with updated PRISMA guidelines (2020 PRISMA statement) (17), and has been registered through PROSPERO (CRD42024555603).

#### 2.1.1 Inclusion criteria

1. Publications documenting preclinical mice models of any type of cancer.
2. Studies that included assessment of traditional ACs (heparin and warfarin) and/or NOACs relating to cancer.
3. Articles published in the English language.
4. Studies assessing single AC groups regardless of other treatment arms.
5. No limit on date of publication.

#### 2.1.2 Exclusion criteria

1. Any study that included nanoparticles-based heparin, heparin-based multidrug delivery system, heparin-based hydrogel, heparin analogue or combinative treatment groups.
2. Any studies relating to *in-vitro* work, human patients, *in silico* studies, or *in vivo* studies relating to animals other than mice.
3. Conference reviews, letters to editor, short communications, abstracts, book chapters and unpublished thesis.

### 2.2 Data sources and search strategy

Medline (Ovid), Embase (Ovid), Web of science, and Scopus databases were selected for this systematic review. The keywords for our database search were stratified into three domains: study sample, disease/condition, and medications including (mice or mouse or murine AND cancer or neoplasia or tumour or tumor or malignancy AND anticoagulant or anti-coagulant or heparin or DOAC or apixaban or dabigatran or rivaroxaban or edoxaban). mp. Search with animal limit was applied to all databases to retrieve specific studies related to our subject of choice, mice. The citations obtained from the database search were imported into Covidence (Melbourne, Australia) and all duplicates and ineligible records were removed before screening by automation tools and manually.

### 2.3 Study selection process

An electronic search for appropriate studies within the defined databases was established by two independent reviewers (H.A and S.H) on 22.9.2022. Conflicts between reviewers were solved by discussion between reviewers (H.A and S.H) and by a third investigator (A.C).

Two independent reviewers (H.A and S.H) extracted data from the studies meeting our inclusion criteria using a standardized data extraction form created in excel. The excel format included information regarding study characteristics (authors, year of publication and country of author origin), animals characteristics (mouse strain, age, weight, and gender), sample size, cancer induction method, anticoagulant treatment characteristics (type of AC, dose, route of administration, timing relative to tumour cell inoculation and duration of administration) and the effectiveness of these ACs on cancer initiation, progression, and metastasis. We also captured any reported complications relating to ACs administration in the included studies.

### 2.4 Statistical analyses

Data were exported into Microsoft® Excel® for Microsoft 365 MSO (Version 2403 Build 16.0.17425.20176) and descriptive analyses were performed. Absolute percentage inter-rater agreement and Cohen's kappa coefficient were calculated using IBM Statistics (SPSS).

Risk of bias was assessed for all the included studies using Office of Health Assessment and Translation (OHAT) risk of bias tool for animal studies. It includes eleven Risk-of-bias domains that are grouped under 6 types of bias (selection, confounding, performance, attrition/exclusion, detection, and selective reporting).

A meta-analysis was performed only if there were studies with similar comparisons reporting the same outcome measures. Mean differences were combined for continuous data and odds ratios for dichotomous ones, using either fixed-effects models or, in the presence of heterogeneity between the studies or paucity of included studies (less than 5), random-effects models. Moreover, in case of a high degree of heterogeneity, the data were explored further to determine if they should be excluded from the meta-analysis. For each meta-analysis a forest plot was created to illustrate the effects of the different studies and the global estimation. In case of meta-analysis performed with fixed effects model a funnel plot was created with the aim of depicting publication bias. Review Manager 5 was used to perform all analyses. The significance cut-off was set at *p*-value < 0.05. Moreover, each meta-analysis underwent a further analysis with the aim of correcting them for the presence of alpha and beta errors, as well as for assessing the power of the analysis. For the abovementioned scope, the authors used the Trial Sequential Analysis (TSA) software (version 0.9 beta, http://www.ctu.dk/tsa). TSA software gave the possibility to calculate the required information size (RIS), the alpha-spending function, the trial sequential monitoring boundaries for benefits and harms, and the futility boundaries. All data collected from the included studies were entered into the TSA software, the alpha error was set at 0.05 and the beta error at 20%. The results of the TSA analysis are presented as a graph with a cumulative z-curve and its relationship with the other curves (trial sequential monitoring boundary, futility boundary and the RIS threshold).

The chi-square test was used to determine if the variation between studies was due to heterogeneity rather than chance. Heterogeneity was assessed using Review Manager 5 (RevMan current version: 5.3.5). Cochrane's test for heterogeneity, which is considered significant at a probability value of less than 0.1, and the *I*^2^ statistic, which measures inconsistency, were used to detect any discrepancies in the estimates of the treatment effects among the studies. A value of *I*^2^ over 50% typically indicates high heterogeneity and relevant inconsistency. A fixed-effect model was applied when heterogeneity among studies was reasonably low (*I*^2^ less than 30%), otherwise a random-effect model was used in the other studies. For the assessment of the mean difference or odds ratio between groups, a fixed-effects model was used or, in case of not negligible heterogeneity >50%, a random effect model was used. In the case of a fixed effects model a funnel plot was used to assess the publication bias.

## 3 Results

**Figure 1:**
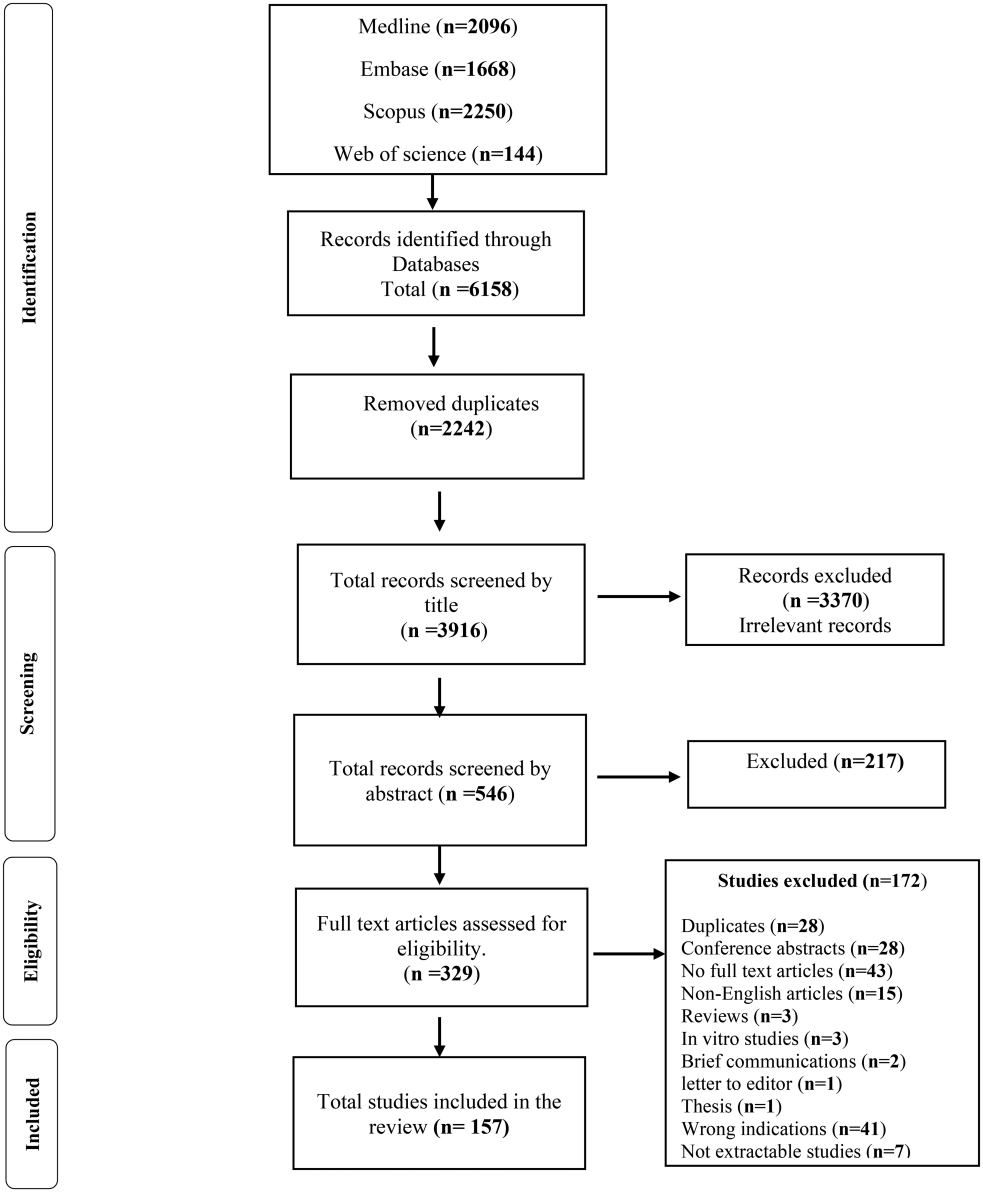
Flow chart showing the process of search and elimination involved in preparing the systematic review for the anticancer effects of anticoagulants in pre-clinical murine models of human and murine cancer.

A total of 6,158 articles were identified through initial database searches. These citations were imported into Covidence, and duplicates (*n* = 2,242) removed before establishment of a screening process (Figure 1). A total of 3,916 records were screened by title and after resolving conflicts between reviewers, the excluded records totalled 3,370. The probability of agreement between reviewers was *p_o_* = 94.91% [CI] with a Cohen's kappa of 0.782, indicative of substantial agreement.

Total records screened by abstract numbered 546, with the probability of agreement between reviewers *p_o_* = 96.7% and Cohen's kappa = 0.931, almost perfect agreement. Records selected for full text article screening totalled 329, with 172 excluded from further analysis for various reasons (Figure 1). The probability of agreement between reviewers was *p_o_* = 98.1%, with Cohen's kappa = 0.961, again indicative of almost perfect agreement. Finally, 157 records were deemed suitable to be included in our study and for initiation of the data extraction process, (Figure 1).

### 3.1 Characteristics of studies meeting inclusion criteria

A total of 157 independent studies were included for data extraction. Studies were published between 1952 and 2022 from 30 different countries: USA (*n* = 51), South Korea (*n* = 21), Germany (*n* = 13), Japan (*n* = 13), China (*n* = 12), UK (*n* = 10), Netherlands (*n* = 8), Italy (*n* = 8) and others (*n* = 41). All studies were conducted in preclinical murine models, among these studies 135 used heparin and its derivatives, 20 reported warfarin and 10 studies utilized NOACs. Occasionally, studies tested multiple AC types at the same time. Studies were conducted using different 50 human cancer cell lines and 127 mouse cancer cell lines and other cell lines with non-reported origin.

Studies conducted using various cancer models including Melanoma (*n* = 51), breast cancer (*n* = 34), lung carcinoma (*n* = 29), squamous cell carcinoma (*n* = 13), sarcoma (*n* = 12), colon cancer (*n* = 10) and types of cancer (*n* = 46). Cancer was induced via allograft tumour (syngeneic mouse models) in 97 experiments, followed by cell line-derived xenograft (CDX) in 60 experiments. Chemically induced models were utilized in three studies, and patient-derived xenografts (PDX) were utilized in 6 studies, while nine studies did not report their method. It is worth mentioning that some studies used more than one type of cancer induction method. Studies were performed utilizing multiple murine strains, the six most common strains utilized in these studies, from most to least common, were C57BL/6, Balb/c, nu/nu, CBA, SCID and C3H/Ne.

#### 3.1.1 Heparin and derivative cancer studies

Heparin and its derivatives (heparin (UFH), Low molecular weight heparin (LMWH), conjugated heparin and non-anticoagulant heparin and other derivatives) were used in 135 studies, including: heparin (UFH) (*n* = 68); Low molecular weight heparin (LMWH); studies that didn't specified the name of drug (*n* = 11); tinzaparin (*n* = 14); dalteparin (*n* = 7); enoxaparin (*n* = 7); nadroparin (*n* = 6); and other LMWHs. Other heparin derivatives included heparin conjugates (*n* = 23) and non-anticoagulants heparin derivatives (*n* = 29).

Multiple routes of ACs administration were performed. The most common was subcutaneous (*n* = 49, 36%), followed by intravenous (*n* = 28, 21%). The duration of AC treatment was extensive and ranged from 1 to 140 days (mean ± SD = 17.1 ± 16.13 days). There was also variability relating to ACs dosing schedules for the various heparin derivatives (i.e., dose adjusted by weight vs. not weight-adjusted) and the frequency of administration was similarly varied (i.e., single vs. multiple doses, once a day, twice a day, etc.). Our findings in relation to the anti-cancer effects of heparin and its derivatives are summarised (Tables 1, 2). In most cases, heparin appears to have anti-metastatic effects in preclinical mice models. While different types of LMWH have been utilized in these studies, they demonstrated varying effects. For example, tinzaparin appears more potent than other LMWHs in terms of reduction of tumour growth and metastasis. Nadroparin and Fraxiparine appear to have no effects on tumour growth, while dalteparin and enoxaparin showed inconclusive results in terms of reduction of tumour growth. On the other hand, non-anticoagulants derivatives of heparin showed promising anti-cancer effects, specifically sulfated-non-anticoagulant heparin (S-NACH) as demonstrated in Table 3.

**Table 1:** Effects of heparin and derivatives on tumour growth and metastasis in preclinical murine models of cancer.

Drug	Studies *n*	Exp *n*	Effect on primary tumor growth *n* = number of exp/study				Effect on metastasis *n* = number of exp/study				Summary conclusions
Heparin (18–84)	68	137	No effect	↓	↑	*N/R or #N/E	No effect	↓	↑	N/R or N/E	Heparin reduces metastasis
			25	30	1	81	32	71	1	33	
LMWH studies (Didn't specified the name of LMWH) (81, 82, 85–93)	11	16	No effect	↓	↑	N/R	No effect	↓	↑	N/R or N/E	LMWH reduces metastasis
			6	4		6	1	3		12	
Tinzaparin (42, 54, 73, 94–104)	14	24	No effect	↓	↑	N/R	No effect	↓	↑	N/R	Tinzaparin reduces tumor growth and metastasis
			2	10		12	5	12		7	
Dalteparin (45, 76, 97, 105–108)	7	11	No effect	↓	↑	N/R	No effect	↓	↑	N/R or N/E	Dalteparin inconclusive effect on tumor growth, but it reduces metastasis
			3	3		5	1	6		4	
Nadroparin (58, 109–113)	6	7	No effect	↓	↑	N/R	No effect	↓	↑	N/R	Nadroparin does not influence tumor growth or metastasis
			4	2		1		1		6	
Enoxaparin (35, 58, 110, 113–117)	7	11	No effect	↓	↑	N/R	No effect	↓	↑	N/R	Enoxaparin is effective in reducing metastasis
			3	3		5	1	7		3	
Drug	Studies (*n*)	Exp *n*.	Effect on primary tumor growth				Effect on metastasis				Summary conclusions
Danaparoid (76)	1	2	No effect	↓	↑	N/R	No effect	↓	↑	N/R	Inconclusive
			1	1						2	
Fragmin (118)	1	1	No effect	↓	↑	N/R	No effect	↓	↑	N/R	Inconclusive
				1						1	
Necuparanib (119)	1	1	No effect	↓	↑	N/R	No effect	↓	↑	N/R	Inconclusive
						1	1				
Fraxiparine (32, 46, 120)	3	12	No effect	↓	↑	N/R	No effect	↓	↑	N/R	No effect
			8	2		2	11			1	
Supersulfated low-molecular weight heparin (ssLMWH) (40, 121)	2	2	No effect	↓	↑	N/R	No effect	↓	↑	N/R	Inconclusive
				1		1		1		1	

*N/R, not reported; #N/E, not extractable explain what this abbreviation means; ↓, reduced; ↑, increased.

**Table 2:** Effects of heparin conjugate on tumour growth and metastasis in preclinical murine models of cancer.

Drug	Heparin derivative conjugates										
	Studies (*n*)	Exp *n*.	Effect on primary tumor growth: (Number of Exp): some studies perform multiple exp				Effect on metastasis (Number of Exp): some studies perform multiple exp				Summary conclusions
LMWH-taurocholate conjugate (LHT7) studies LHTR7 (122–128)	7	12	No effect	↓	↑	N/R	No effect	↓	↑	N/R or N/E	Reduce tumor growth. No available studies on metastasis
			1	10		1				12	
LMWH-taurocholate-tetrameric deoxycholate (LHTD4) studies (127, 129, 130)	3	8	No effect	↓	↑	N/R	no effect	↓	↑	N/R	Reduce tumor growth and metastasis
			1	3		4		5		3	
LHbisD4 (131)	1	2	No effect	↓	↑	N/R	No effect	↓	↑	N/R	Inconclusive
						2		2			
LMWH conjugated with deoxycholic acid (DOCA); LMWH-DOCA, (LHD, (LHD 1.5, 1, 2, 4); and Heparin conjugate with deoxycholic acid (Doc-heparin, HFD 1, 2,3) and (HD) (70, 85, 90, 132–135)	7	23	No effect	↓	↑	N/R	No effect	↓	↑	N/R	Orally active heparin derivatives reduce tumor growth
				19		4		4		19	
Heparin. Folate-HL conjugate (FHL) (136)	1	1	No effect	↓	↑	N/R	No effect	↓	↑	N/R	Inconclusive
				1						1	
Conjugate Heparin-Lithocholic Acid (HL) (70, 136)	2	2	No effect	↓	↑	N/R	No effect	↓	↑	N/R	Inconclusive
				2						2	
(LHsura) (89) A low molecular weight heparin and suramin fragment conjugate	1	1	No effect	↓	↑	N/R	No effect	↓	↑	N/R	Inconclusive
				1						1	
GH1, GH2, GH3 Glucosylated heparin derivative (120)	1	3	No effect	↓	↑	N/R	No effect	↓	↑	N/R	Inconclusive
GH1, GH2				2						2	
GH3 Glucosylated heparin derivative			1							1	

**Table 3:** Effects of non-anticoagulant and low anticoagulant heparin derivatives on cancer in preclinical murine models of cancer.

Drug	Non anticoagulants heparin derivatives										
	Studies (*n*)	Exp (*n*)	Effect on primary tumor growth				Effect on metastasis				Summary conclusions
Sulfated-non-anticoagulant heparin (S-NACH) (42, 95, 96, 137)	4	8	No effect	↓	↑	*N/R	No effect	↓	↑	N/R	S-NACH reduces tumor growth and metastasis
				4		4	1	5		2	
Non-anticoagulant heparin derivatives (NAC); NAC-HCPS, NAC2500, NAC6000, NAC8000, NAC10000, N-desul N-ac Heparin, O/N-desul N-ac heparin derivatives (44, 74, 101, 138)	4	10	No effect	↓	↑	N/R	No effect	↓	↑	N/R	Non-anti-coagulant (NAC) heparin derivatives reduce tumor growth, but it appears to have no effect on metastasis
				2		8	4	4		2	
Heparin adipic hydrazide (HAH) (139)	1	1	No effect	↓	↑	N/R	No effect	↓	↑	N/R	Inconclusive
			1							1	
Non-anticoagulant form of LMWH/NA-LMWH (114)	1	1	No effect	↓	↑	N/R	No effect	↓	↑	N/R	Inconclusive
						1		1			
Low anticoagulant activity heparin derivatives											
SST0001 Roneparstat (100 NA-RO.H.A); 100% N-acetylated and 25% glycol-split heparin studies (140–142)	3	9	No effect	↓	↑	N/R	No effect	↓	↑	N/R or #N/E	Roneparstat reduces tumor growth
			1	4		4		1		8	
Carboxyl-reduced heparin (CR-heparin) (41, 55, 84)	3	3	No effect	↓	↑	N/R	No effect	↓	↑	N/R	Low anticoagulant activity, reduced tumor metastasis
						3		3			
Reduced oxyheparin heparin derivatives (RO.H), and (100 NA-RO.H.A) (143)	1	8	No effect	↓	↑	N/R	No effect	↓	↑	N/R	Reduced metastasis in one study
						8	2	6			
LAC-heparin (82)	1	12	No effect	↓	↑	N/R	No effect	↓	↑	N/R	Periodate-oxidized and borohydride-reduced heparin with low anticoagulant activity (LAC heparin). reduced metastasis in one study
						12	2	10			
Butanoylated heparin; (83)	1	3	No effect	↓	↑	N/R	No effect	↓	↑	N/R	O-acylating low molecular weight heparin with butyric anhydride—weak anticoagulant; reduced tumor growth in one study
				3						3	
N-desulfated, 6-O desulfated, 2-O-desulfated heparin, N-desulfated, 2-O,3-O-desulfated heparin, N-desulfated heparin, N-2,3-DS-heparin (84, 144–146)	4	13	No effect	↓	↑	N/R	No effect	↓	↑	N/R or N/E	reduced anti-coagulant activity, reduced metastasis
			1	2		10		9		4	
N-acetylated N-desulfated heparin, N-resulfated N-and O-desulfated heparin (41, 55)	2	4	No effect	↓	↑	N/R	No effect	↓	↑	N/R	Almost devoid of anti-coagulant activity. It reduces metastasis
						4		4			
LABH (93)	1	1	No effect	↓	↑	N/R	No effect	↓	↑	N/R	Low-anticoagulant bovine heparin (LABH): inconclusive
			1							1	
58 NA-H; 58% N-acetylated heparin (58NA-H) (143)	1	6	No effect	↓	↑	N/R	No effect	↓	↑	N/R	Reduced anti-coagulant activity; reduced tumor metastasis in one study
						6	1	5			
Heparin-DOCA (bile acid acylated-heparin derivative (heparin-DOCA) (68, 69)	2	6	No effect	↓	↑	N/R	No effect	↓	↑	N/R	Lower anticoagulant activity, reduced tumor growth and metastasis
				3		3		3		3	

*N/R, not reported; N/E not extractable; ↓, reduced; ↑, increased.

#### 3.1.2 Warfarin cancer studies

Warfarin was evaluated in 20 studies meeting our selection criteria. Warfarin was tested on different types of cancer in murine models, including lung carcinoma (6 out of 20 studies), sarcoma and fibrosarcoma (7 out of 20 studies), breast carcinoma (4 out of 20 studies) and other types of cancer, including bladder cancer, melanoma and neuroblastoma. The cancer cell line utilized in 18 of these studies was of murine origin, while only two studies used human cancer cell lines. The most common route of administration was via the drinking water (14 out of 20 study), with the dose ranging from 0.5 mg/L to 9.4 mg/L (average of 5.6 mg/L). Treatment duration ranged from 1 to 28 days with mean ± SD (11.16 + −7.25) days as demonstrated in Table 4.

**Table 4:** Effects of warfarin on cancer in preclinical murine models.

Study	Cancer type	Dose schedule; (Dose, frequency, route)	Duration of treatment (Days)	Timing in reference to tumor inoculation^a^	Effect on Primary tumor growth	Effect on metastasis
Baker et al. (147)	Mouse Sarcoma	7.6 mg/L/Daily/DW	13	+9 day	↓	No effect
Biggerstaff et al. (148)	Mouse Neuroblastoma	3.5 mg/L/Daily/DW	14	−4 days	↓	N/R
Brown (149)	Mouse Sarcoma	7.5 mg/L, 5 mg/L/daily/DW	7	−4 days	N/R	↓
	Mouse breast cancer	7.5 mg/L, 5 mg/L/daily/DW	7	−4 days	N/R	↓
Carmel and Brown. (150)	Mouse Sarcoma	N/R/daily/DW	7	−4 days	N/R	↓
Colucci et al. (37)	Mouse Lewis lung carcinoma	7.5 mg/L, 1.5–2 mg/L,/Daily/DW	N/R	N/R	N/R	↓
Dumont et al. (151)	Mouse Lewis lung carcinoma	3 mg/kg/every two days/oral (NOS)	14	+24 h	No effect	No effect
	Mouse Lewis lung carcinoma	3 mg/kg, every two days, oral (NOS)	14	−7 days	No effect	↓
Fasco et al. (152)	Mouse Lewis lung carcinoma	4.8 µg/ml, 2.4 µg/ml, daily/DW	N/R	N/R	↓	↓
Ghersa and Donelli (153)	Mouse Lewis lung carcinoma	5 mg/L, 1 mg/L/daily,/oral (NOS)	N/R	+13 days	No effect	No effect
Gorelik (38)	Mouse Melanoma; Madison lung carcinoma	8 µg/ml/daily/DW	2	−2 days	N/R	↓
Gorelik (43)	Mouse Melanoma; Madison lung carcinoma	8 mg/L, daily/DW	2	−2 days	N/R	↓
Ketcham et al. (154)	Human Bladder cancer	9.1 mg/L, 9.4 mg/L, Daily/N/R	N/R	N/R	N/R	↓
	Breast Adenocarcinoma	9.1 mg/L, 9.4 mg/L,/Daily/N/R	N/R	N/R	N/R	↓
	Fibrosarcoma	9.1 mg/L, 9.4 mg/L, Daily/N/R	N/R	N/R	↓	↓
Ocal et al. (155)	Pancreatic ductal adenocarcinoma	0.2 mg/kg/5 days a week, N/R	14	N/R	No effect	N/R
Lione et al. (29)	Mouse melanoma	1.5 mg, 2 mg, 4 mg/Once a day/ip	2	−24 h, −48 h	N/R	↓
Lorenzet et al. (156)	Mouse Fibrosarcoma	7.5 mg/L, 1–2.5 mg/L/N/R/DW	19	+N/R	N/R	No effects
Maeda et al. (157)	Mouse melanoma	0.03 mg/kg	1	−6 h	N/R	No effect
		0.1 mg/kg			N/R	No effect
		0.33 mg/kg			N/R	↑
		1 mg/kg/once a day/Oral gavage			N/R	↑
Kirane et al. (15)	Human pancreas cancer Pan02	0.5 mg/L daily/DW	14–28	+when tumor visible by ultrasound (∼10 mm^3^)	↓	↓
	Human pancreas cancer KIC	0.5 mg/L/daily/DW			↓	↓
	Human pancreas cancer Panc-1	1 mg/L/daily/DW			No effect	No effect
	Human pancreas cancer AsPC-1	1 mg/L/daily/DW			No effect	↓
	Human pancreas cancer Capan-1	1 mg/L/daily/DW			No effect	No effect
	Human pancreas cancer Panc-1	1 mg/L/daily/DW	N/R	−48 hr	N/R	↓
	Human pancreas cancer Panc-1	1 mg/L/daily/DW	N/R	+48 hr	N/R	↓
Seth et al. (97)	Murine colon carcinoma CT26LacZ	0.000266 g/N/R/DW	N/R	−3	N/R	↓
				−1		
				+1		
Ryan et al. (158)	Autochthonous tumours	9.10 mg/L/N/R/DW	N/R	N/R	N/R	↓
Ryan et al. (159)	Mammary adenocarcinoma	9.325 mg/L/N/R/DW	15	N/R	N/R	↓
	anaplastic sarcoma T241	9.215 mg./L./N/R/DW	21	N/R	N/R	↓
Ryan et al. (160)	Sarcoma T241	9.235 mg/L/N/R/DW	10	+2 days	N/R	↓
	Mammary adenocarcinoma	9.215 mg/L/N/R/DW	12	+2 days	N/R	↓

^a^ Timing of the warfarin administration given in reference to injection of tumour cells. A negative value refers to NOAC administration prior to tumour inoculation/induction, ↓, reduced; ↑, increased; *N/R, not reported; N/E, not extractable; DW, drinking water; ip, intraperitoneal.

#### 3.1.3 NOAC cancer studies

NOACs, including dabigatran and rivaroxaban, were investigated in 10 studies meeting our selection criteria. For dabigatran, the most common route of administration was oral, either gavage or chow diet. For administration of dabigatran by oral gavage, the average dose was 80.6 mg/kg (45–120 mg/kg), and via chow diet it was consistent across all studies at 10 mg/g of chow. In most studies, duration of dabigatran administration was not clearly defined and therefore not extractable, however treatment duration ranged from 10 to 28 days (average 19.6 ± 9.07 days). The effects of dabigatran on tumour growth and metastasis in relation to timing of administration were variable (no effect/decreased tumour growth and metastasis not recorded) and not consistent across the studies evaluated, albeit with only two available (Table 5). Murine cancer cell lines were utilized in 6 out of 8 studies in the dabigatran group, with two studies utilized human cancer cell lines, both being breast cancer cell lines. Dabigatran showed controversial results in these two studies against human breast cancer; in one study, it had no effect, while in the other it reduced metastasis.

**Table 5:** Summary of studies assessing the effects of NOAC on cancer in pre-clinical murine models.

Study	Drug	Cancer type	Tumor model: site of cell inoculation	Dose schedule; (Dose, frequency, route)	Duration of treatment (days)	Timing in reference to tumor inoculation^a^	Effect on Primary tumor growth	Effect on metastasis
Alexander et al. (161)	Dabigatran etexilate	Mouse mammary adenocarcinoma 4T1	Orthotopic	80 mg/kg/twice/day/weekdays/oral gavage; 120 mg/kg once/day/weekend/oral gavage	^#^N/E	+2 weeks	No effect	*N/R
Alexander et al. (162)	Dabigatran etexilate	Mouse ovarian cancer ID8-luc	Ectopic	80 mg/kg twice a day/weekdays/oral gavage; 10 mg/g once/day/weekend/food	28	+5 weeks	↓	N/R
Buijs et al. (106)	Rivaroxaban	Human breast cancer MDA-MB-231	Orthotopic	0.4 mg/g chow/daily	N/E	N/R	No effect	No effect
	Dabigatran etexilate			10 mg/g chow/daily	N/E	N/R	No effect	No effect
	Rivaroxaban			0.4 mg/g chow/daily	N/E	N/R	No effect	No effect
	Rivaroxaban			0.4 mg/g chow/daily	N/E	N/R	No effect	No effect
	Rivaroxaban			1 mg/g chow/daily	N/E	N/R	No effect	No effect
	Dabigatran etexilate			10 mg/g chow/daily	N/E	N/R	No effect	No effect
DeFeo et al. (163)	Dabigatran etexilate	Mouse mammary carcinoma 4T1	Orthotopic	45 mg/kg twice/day, weekday/oral gavage; 60 mg/kg, once/day/on weekend oral gavage	N/E	−1 day	↓	↓ liver metastasis
								No effect on lung metastasis
Feinauer et al. (98)	Dabigatran etexilate	Human breast cancer Jimt1	Ectopic	80 mg/kg/oral gavage/twice/day	10	−2 days	N/R	↓
Graf et al. (107)	Rivaroxaban	Fibrosarcoma T241	Ectopic	0.4 mg/g chow/daily	N/E	+14 days	↓	↓
Peraramelli et al. (164)	Dabigatran etexilate	Mouse, N/R, melanoma B16	Ectopic	N/R/N/R/food	N/R	−4 days	↓	↓
	Dabigatran etexilate				N/R	−4 days	No effect	N/E
	Dabigatran etexilate				N/R	−4 days	No effect	N/E
	Dabigatran etexilate	Mouse melanoma YUMM3.1	Ectopic	N/R/N/R/Food	N/R	−4 days	↓	No effect
Maqsood et al. (165)	Rivaroxaban	Human pancreas BxPc-3	Ectopic	0.5 g/kg chow/N/R/food	N/R	+When tumours reached a mean volume of ∼100 mm^3^	No effect	N/R
	Rivaroxaban	Human pancreatic cancer MIA PaCa-2	Ectopic	0.5 g/kg chow/N/R/food	N/R	+ When tumours reached a mean volume of ∼100 mm^3^	No effect	N/R
Smeda et al. (166)	Dabigatran etexilate	Mouse mammary adenocarcinoma 4T1-luc2-tdTomato	Ectopic	100 mg/kg/twice/day (weekday), once daily (weekend)/oral gavage	N/E	−3 days	N/R	↑
Shi et al. (167)	Dabigatran etexilate	Murine pancreatic Panc02	Orthotopic	80 mg/kg/twice daily/oral gavage	21	+7 days	No effect	↑

^a^ Timing of the NOAC administration given in reference to injection of tumour cells. A negative value refers to NOAC administration prior to tumour inoculation/induction, ↓, reduced, ↑, increased. *N/R, not reported; N/E, not extractable.

Murine breast cancer models were utilized in 3 studies of the dabigatran group in which dabigatran's effect ranged from no effect to an increase in the metastatic burden.

Rivaroxaban was evaluated in three studies in the literature. It was administrated via chow diet in all studies (0.4–0.5 mg/g chow) and had no effect in almost all studies on tumour growth and metastasis (Table 5). This may be related to timing of administration which was post tumour inoculation in all studies (Table 5). Rivaroxaban was tested in two murine model of cancer of human cell origin including human breast cancer and human pancreatic cancer. It showed no effect on tumour growth and/or metastasis in both models.

#### 3.1.4 Primary and secondary outcomes

Across all studies the primary outcomes in our systematic review included the effects of AC on tumour growth (volume and weight) and tumour metastasis (incidence of metastasis, number of nodules, site, and staging). The secondary outcome pertained to the optimal dosage of AC, the most common route of administration, the duration of AC treatment in murine preclinical models of cancer, and the timing of administration in relation to tumour inoculation (human and murine). Additionally, survival rate, mortality, complications associated with administration of AC in mouse models were reported.

### 3.2 Study quality and risk of bias

**Figure 2:**
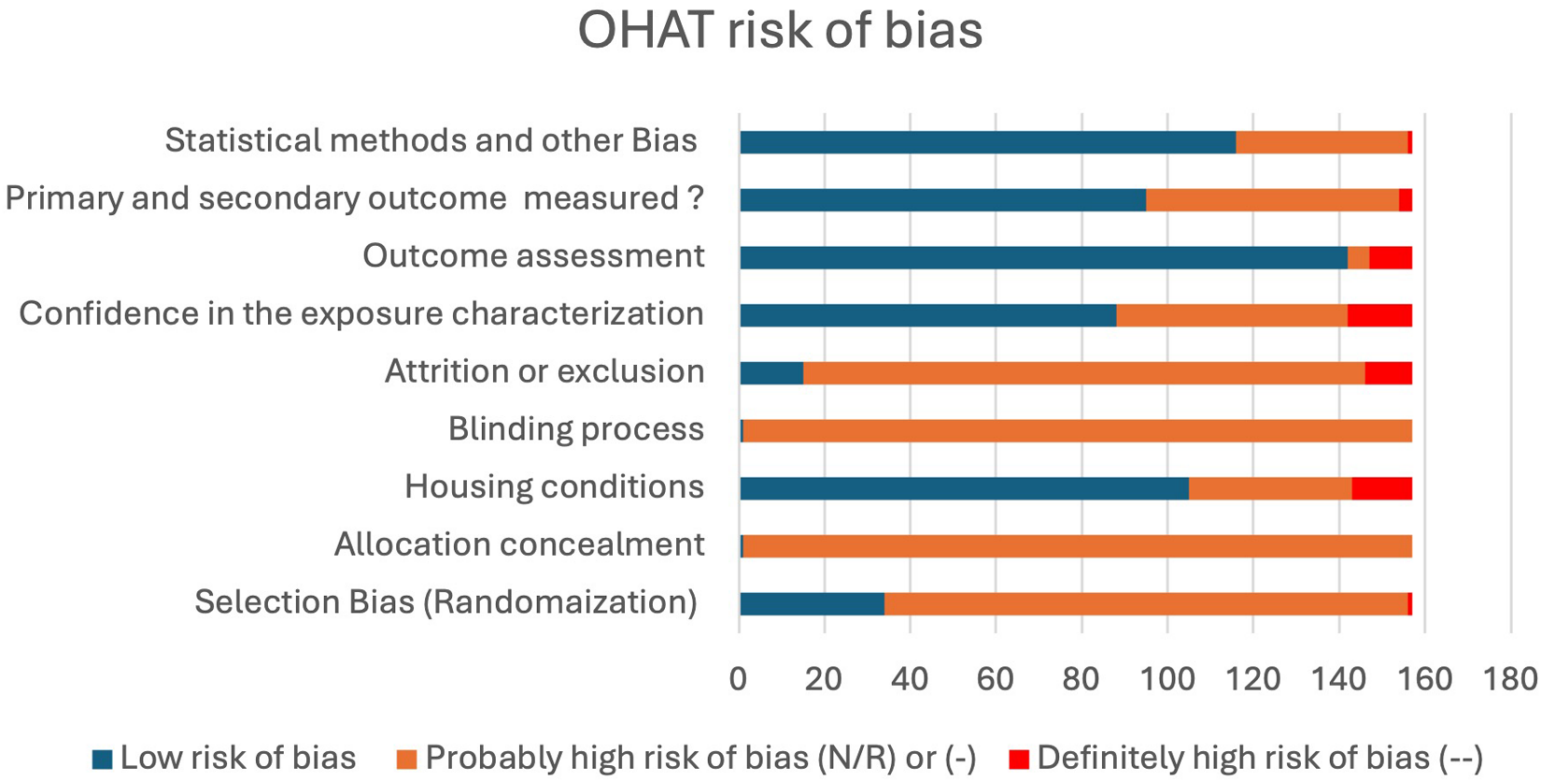
Bar chart of the probability of bias among reviewed studies. (N/R) or (-), indirect evidence of high risk-of-bias practices or there is insufficient information. (--), direct evidence of high risk-of-bias practices. Number of studies (*n* = 157).

Risk of bias was assessed utilizing Office of Health Assessment and Translation (OHAT) risk of bias tool for animal studies. Two reviewers independently conducted the risk of bias assessment (HA and FA). All studies, except one were probably at high risk of bias (N/R) for at least two criteria. These studies failed to report the allocation concealment process and the blinding process involved in exposure given to animals (Figure 2). Reviewed studies (60.5%) reported successfully the primary and secondary outcomes, while 39.4% reported with insufficient information about selective outcomes. Fifty six percent of the studies showed a confidence in exposure characteristics by performing the anticoagulant activity tests for the administered drugs; while 43.9% failed to report information regarding coagulation test or stability of the compounds used. In most studies (90.4%), the outcomes were assessed utilizing well established methods in the literature, while 9.6% failed to report the method of assessment. There was no clear method of animal randomization to treatment or control groups in 78.3% of the studies, while only 21.6% reported randomization of animals in these studies. The majority of the studies (90.4%) did not report the survival or mortality rate of animals in general and only 9.5% of studies reported the survival statistics of experimental animals (Figure 2).

### 3.3 Meta-analysis

We conducted a meta-analysis on a subset of studies in this systematic review. Meta-analysis was performed only on studies with a homogeneous design, using Review Manager 5.3 (RevMan) software with a significance cut-off value set at *p* < 0.05. A forest plot was used to show the results of the meta-analysis and the contribution of the individual studies along with the global estimation. The meta-analysis excluded all studies that solely reported percentage of metastasis inhibition without providing additional information regarding the event or total event. This exclusion was necessary since such percentage data lacked clarification regarding whether the study referred to the number of animals with metastasis or to the percentage of metastasis extent per animal. Moreover, all studies reporting data in a graphical format were excluded, since such data was deemed not extractable.

We divided anticoagulants in to four main categories and three comparisons:

1. Warfarin vs. control
2. Heparin & derivatives vs. control
3. Non anticoagulant heparin vs. control
4. Direct oral anticoagulants vs. control

In each comparison, 3 outcomes (when possible) were evaluated:

a. Metastasis formation (expressed as %)
b. Metastasis formation (expressed as number of colonies)
c. Tumour weight or volume

Initially 50 studies were selected for further analysis. Subsequently, because of insufficient studies in each category, we performed metanalysis on 41 out of 157 studies; thirty one student on heparin and its derivatives (18–36, 85, 86, 94, 95, 105, 109–111, 122, 168–170) and ten warfarin studies (29, 37, 147, 149, 153, 154, 156, 158–160). All studies with an undefined number of participants were excluded and meta-analysis with less than 3 included studies in each category was not performed. For that reason, non-anticoagulants heparin studies were excluded from further analysis. For NOACs, there was only a single study, which hindered meta-analysis for this group.

#### 3.3.1 Heparin and its derivatives metanalysis results

**Figure 3:**
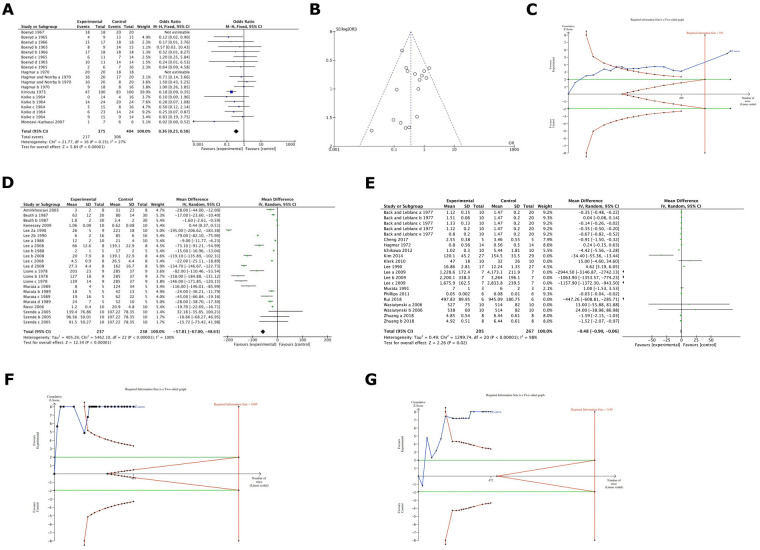
**(A)** Cumulative meta-analysis of heparin and derivatives effect on cancer metastsis formation (%) in the included studies. Results were statistically significant in favour of Heparin & its derivatives. (with a factor of around 36%). Odds ratio was 0.36. **(B)** A Funnel Plot (FUNNEL PLOT 3) for the analysis of the publication bias among heparin studies. **(C)** TSA analysis of heparin group and its derivatives demonstrated that the z-curve crosses both the alpha-spending function and the conventional boundary and also reaches the RIS threshold. **(D)** Cumulative meta-analysis of heparin and its derivatives effects on cancer in terms of metastasis formation inhibition (expressed as number of colonies) in the included studies. It deals with comparison 2 outcome b: statistically significant in favour of heparin & derivatives (the mean difference of number of colonies is around 58). **(E)** Cumulative meta-analysis of heparin and its derivatives effects on tumour growth (volume or weight) in the included studies. It demonstrated the comparison 2 outcome c. Results were statistically significant in favour of heparin and its derivatives, the difference of tumour weight or volume is around 0.48. **(F)** TSA analyses of heparin group and its derivatives depicted that, even if the z-curves cross both the alpha-spending function and the conventional boundary, they do not reach the RIS threshold. **(G)** TSA analyses of heparin group and its derivatives depicted that, even if the z-curves cross both the alpha-spending function and the conventional boundary, they do not reach the RIS threshold.

Our analysis of studies relating to heparin and its derivatives showed statistically significant results in favour of heparin and its derivatives in terms of metastasis formation inhibition (%) with a factor of 36%, Odds ratio was 0.36 (95% CI: 0.25–0.50) (Figure 3A). Since heterogeneity among studies was low enough (27%) a fixed effect model was used, and a Funnel Plot was performed (Figure 3B) for the analysis of the publication bias. Such evidence is considered to have high power as the TSA analysis demonstrated that the z-curve crosses both the alpha-spending function and the conventional boundary, as well as reaching the RIS threshold (Figure 3C). Additionally, results were statistically significant in favour of heparin and its derivatives in terms of metastasis formation inhibition (expressed as number of colonies). In this case the mean difference is 57.81 (Figure 3D). Moreover, in terms of tumour weight or volume inhibition, results were statistically significant in favour of heparin and its derivatives, the mean difference of tumour weight or volume is 0.48 (Figure 3E). Nevertheless, it should be noted that the evidence retrieved by the last two meta-analyses cannot be considered enough reliable as the TSA analyses depicted that, even if the z-curves crossed both the alpha-spending function and the conventional boundary, they do not reach the RIS threshold (Figures 3F,G).

#### 3.3.2 Warfarin metanalysis results

**Figure 4:**
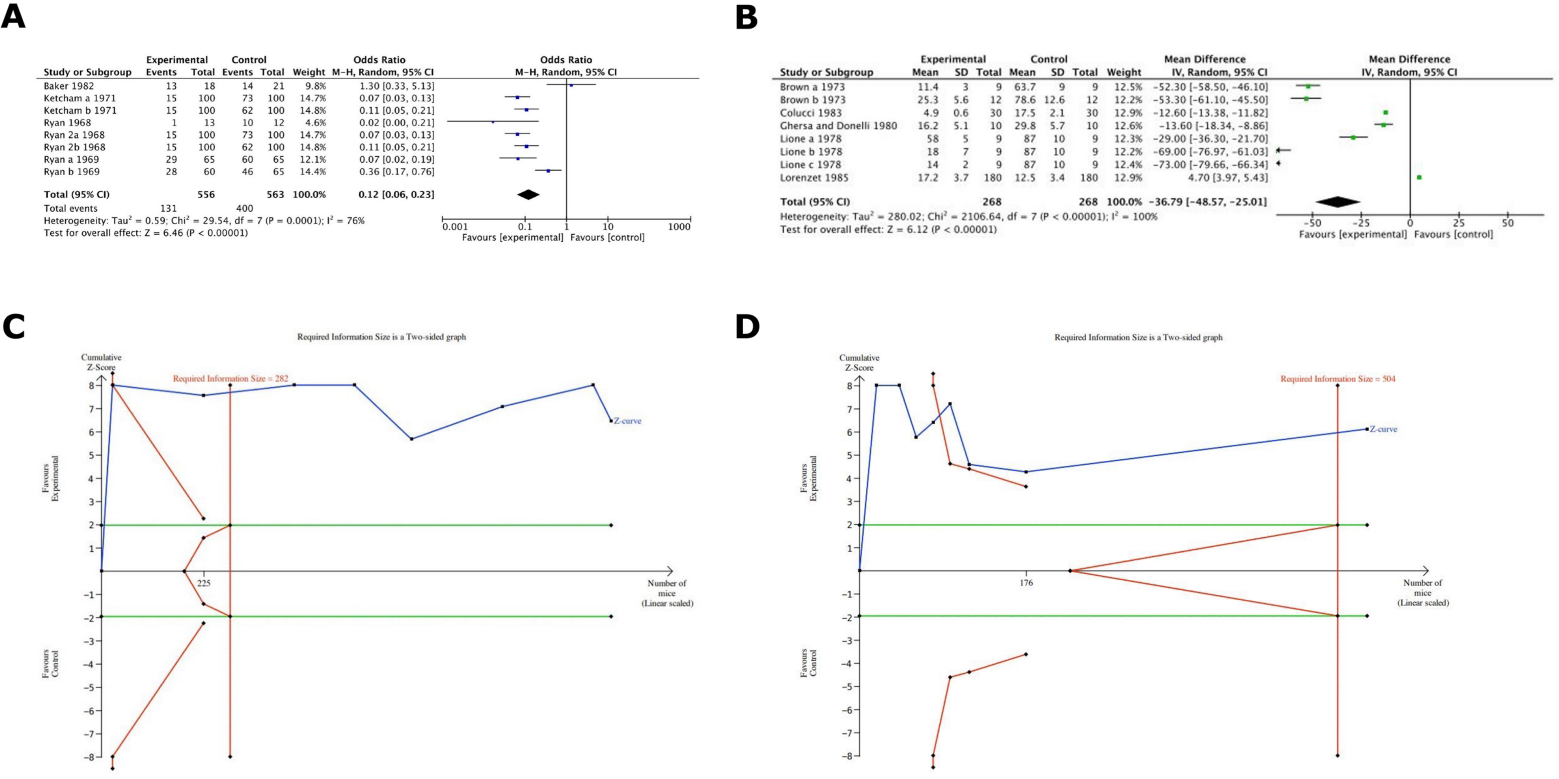
**(A)** Cumulative meta-analysis of warfarin on cancer metastsis in the included studies. It demonstrated the comparison 1 outcome a: statistically significant in favour of warfarin (with a factor of around 100%). **(B)** Cumulative meta-analysis of warfarin effects on cancer metastasis in the included studies. It showed comparison 1 outcome b: statistically significant in favour of warfarin (the difference of number of colonies is around 37). **(C)** TSA analysis for warfarin groups depicted that the z-curve crosses both the alpha-spending function and the conventional boundary and also reaches the RIS threshold. **(D)** TSA analysis for warfarin groups depicted that the z-curve crosses both the alpha-spending function and the conventional boundary and also reaches the RIS threshold.

Results were statistically significant in favour of warfarin (with a factor of 100%) in terms of metastasis formation inhibition, Odds ratio 0.12 (95% CI 0.06–0.23) (Figure 4A). Further, results demonstrated statistically significant metastasis formation inhibition in favour of Warfarin with a mean difference of number of colonies being 36.7 (Figure 4B). All evidence regarding Warfarin was considered to have high power as in both cases the TSA analysis depicted that the z-curve crosses both the alpha-spending function and the conventional boundary while also reaching the RIS threshold (Figures 4C,D).

## 4 Discussion

Anticoagulants (ACs) are frequently prescribed medications for patients at high risk of developing blood clots. The anti-inflammatory and anticancer effects of anticoagulant therapy in patients with malignancies have been outlined in a recent review on this topic (171). However, to date, only the anti-inflammatory effects have been documented in studies involving human participants (171).

Recently, studies have shown that ACs also improve survival of cancer patients. A systematic review of 29 studies relating to warfarin and heparin explored their effects on cancer patient survival, revealing that warfarin may improve patient survival and it may reduce the risk of urogenital cancer, while LMWH improved the survival of patients with small cell lung cancer (172). In a similar vein, a study retrospectively assessed 1,486 patients diagnosed with primary gastric cancer (GC) who underwent radical resection. Among these patients, 34.5% received postoperative anticoagulation therapy (AC), and the findings indicated that anticoagulation therapy after radical gastrectomy can significantly enhance the overall survival of GC patients, while those who did not receive AC exhibited reduced overall survival (173).

Contradictory evidence from a further systematic review (9 studies, 5,987 patients, 98.4% with advanced-stage disease) reported no survival benefit of LMWH in cancer patients (174). An additional systematic review assessing LMWH on survival outcomes of patients with solid tumours (45 randomized clinical trials studies) showed that LMWH treatment failed to improve survival of patients with malignancy (175). Pertinently, our unpublished data demonstrate that oral cancer patient survival in those treated with chemotherapy and simultaneously receiving anticoagulant therapy had their survival reduced by half. Overall, there is no conclusive evidence for ACs influencing cancer outcomes and additional research is needed to determine whether this experimental evidence could influence patient prognosis and overall survival rates (171). Therefore, we a performed a first systematic review and meta-analysis of Acs (both traditional and NOAC) in preclinical cancer research using human and murine cancers and conducted in murine models.

The anti-cancer effects of heparin were first reported in animals in 1931 by Goerner (176). Their anti-metastatic properties can be attributed to various factors, including inhibition of the heparanase enzyme, mainly involved in cancer progression; inhibition of angiogenesis, lymphogenesis, and P-selectin-mediated platelets-cancer cell adhesion (96), Additionally, heparanase may enhance the recognition of the cancer cell by NK cells and enhance cancer clearance (25). In most studies, heparin showed anti-metastatic effects when administrated before tumour cell inoculation, but it had no further anti-tumour effects after this stage (38). This may be attributed to its inhibitory effect on P selectin. There appears to be a synergistic effect of P and L selectins in facilitating metastasis as demonstrated in murine research. L-selectin-deficient (*L^−/−^*) mice showed a significant reduction in metastasis highlighting the role of L-selectin in facilitating metastasis; therefore, heparin administered at early time point before tumour inoculation acts by inhibiting P selectin (platelets-tumour interaction) while when administered in a later time after tumour inoculation, heparin acts on L selectin on leukocyte, NK, monocyte (39, 177).

In this systematic review, we identified 4 main types of heparin and heparin derivatives including un-fractioned heparin (UFH), low molecular weight heparin (LMWH), conjugated heparin and non-anticoagulant heparin and other derivatives in most heparin studies, heparin was shown to have anti-cancer effects in different cancer models and in terms of reducing primary tumour growth and metastasis (Table 1). In some studies, the percentage of metastasis inhibition by heparin was impressive, ranging from 74% to 94.3% (31, 40).

Since heparins side-effect of bleeding limits its use in preclinical murine studies, heparin derivatives that have a high antiangiogenic properties and low anticoagulant effects have been created. LMWH-taurocholate conjugate (LHT7) has been introduced as a heparin conjugate with a 100 times binding affinity to angiogenic growth factor VEGF compared to LMWH. We also identified 7 studies that showed that LHT7 reduced tumour growth in preclinical murine models (123–126). However, since there is a requirement for frequent parental injection of LHT7 and low oral bioavailability, an oral active heparin conjugated to tetrameric deoxycholic acid (DOCA) has more recently formulated (LHTD4). The effects of LHTD4 on cancer were assessed in 3 studies, which similarly showed a reduction of tumour growth and metastasis (125, 127). LHTD4 was evaluated in three types of cancers, including an ectopic murine SCC7 model, an orthotopic human and murine breast cancer model, and in ectopic human lung cancer model. In all these studies LHTD4 was administrated orally and after tumour inoculation, the most common utilized dosages were 5 and 10 mg/kg. LHTD4 inhibited tumour growth in mice model (ranging from 73% to 56.8%) (125, 127).

A non-anticoagulant heparin or derivative with low anticoagulant activity was formulated by selective desulfation, removing sulfated groups from the antithrombin binding region (ATBR). This non-anticoagulant heparin that retained other biological activities can be produce by periodate oxidation such as Glycol-split heparins (178). We retrieved 29 studies using these compounds to treat cancer in preclinical murine models. Among such non-AC compounds, sulfated-non-AC heparin (S-NACH); non-AC (NAC) heparin derivatives and low AC heparin derivatives; SST0001 Roneparstat, Carboxyl-reduced heparin (CR-heparin) and desulfated heparin derivatives demonstrated promising results in reducing both tumour growth and metastasis (41, 42, 95, 114, 140) (Table 3).

Despite the fact that warfarin is a veteran drug and the existence of new guidelines for cancer therapy associated thrombosis with NOACs medications, warfarin remains a common treatment strategy for many cancer patients due to its low cost and patient preference (179). Therefore, it is important to determine the effects of warfarin on cancer biology. We assessed 20 articles pertaining to warfarin effects on various types of human and murine cancer in preclinical murine models. Our meta-analysis showed statistically significant in favour of warfarin (with a factor of 100%) in terms of metastasis formation inhibition, as well as metastasis inhibition. Moreover, in most studies, warfarin reduced metastasis specifically when administrated before tumour inoculation (15, 43, 97, 151). While its effect on tumour growth was unconclusive, warfarin was shown to have no effect on primary tumour growth or metastasis in almost all studies when administrated after tumour inoculation. The proposed anti-cancer properties of warfarin may mechanistically involve prevention of fibrin formation around tumour cells circulating in the blood, making these cells more susceptible to clearance by immune cells (22). Moreover, GAS6 (growth arrest–specific 6), the ligand of the AXL receptor tyrosine kinase family is associated with immune regulation and cancer development. Warfarin treatment inhibits AXL receptor signalling, blocking the malignant traits of aggressive carcinoma cells and enhancing anti-tumour natural killer cell activity at doses that do not affect coagulation (180).

Recently, four randomized clinical trials (RCTs) have demonstrated that new oral anticoagulants are good alternatives to LMWH for the acute management of cancer-associated thrombosis, yielding effective and safe outcomes (181–184). Moreover, NOACs have been shown to improve overall survival for patients with head and neck cancer compared to warfarin (185). We retrieved 10 studies in the literature (98, 106, 107, 161–167) that studied the anti-cancer effects of NOACs, including dabigatran and rivoroxaban, in preclinical mice models (Table 5). We found variable findings relating to the impact of dabigatran on tumour growth and metastasis. Dabigatran effects on cancer was purported to be related to its antithrombin properties, since thrombin can enhance tumour progression via fibrin formation and activation of protease-activated receptors (PARs) and platelets. Therefore, dabigatran is likely to be useful in cancer patient (161, 162). Rivaroxaban was tested in 3 studies (106, 107, 165), and in 2 out of 3 studies it showed no effect on tumour growth and metastasis that may be related to timing of administration occurring after tumour inoculation in these studies. These findings align with those of Najidh et al. whose systematic review included 9 studies demonstrating that NOACs had no effects in a xenograft mouse models, while their effects on tumour growth and metastasis in syngeneic mouse models depended on the timing of NOACs administration in relation to tumour inoculation and type of cancer model (9). On the same occasion, a recent study examined Edoxaban, one of the NOACs, and found that it significantly inhibits tumour cell proliferation via the factor Xa-PAR2 (Protease-Activated Receptor 2) pathway, which is activated by coagulation and inflammation in Colon26-inoculated mice, ultimately resulting in tumour cell apoptosis (186).

This study systematically evaluates the effects of anticoagulants in murine cancer models, offering a comprehensive analysis of their therapeutic potential in preclinical research. By synthesizing data from various studies, it aims to provide valuable insights into the role of anticoagulants in cancer treatment, focusing on their applicability in future animal models. Additionally, the study seeks to create a reference tool to facilitate the translation of these findings into clinical settings, contributing to the development of more effective cancer therapies.

However, several limitations must be considered. The meta-analysis included only 41 of the 157 eligible studies due to insufficient data documentation, with much of the relevant information presented graphically, complicating extraction for analysis. Additionally, 90% of the studies on warfarin were over ten years old. Considerable variability and a lack of standardization in the dosage units used for heparin administration across the included murine studies also made it difficult to establish the optimal dosage for different heparin species.

The risk of bias was high for certain criteria, impacting the validity of the conclusions. For instance, only 56% of the studies provided reliable information on treatment characteristics by conducting anticoagulant activity tests on the administered drugs in murine models. Furthermore, only 60.5% of the studies reported sufficient information on selective outcomes. Randomization and allocation procedures were largely absent, which further affects the internal validity of the findings.

Findings from our study will serve as a reference and lay the groundwork for appropriate implementation of anticoagulants in designing future preclinical studies, which, if successful, may contribute to the advancement and design of future cancer therapy combinative trials with ACs. Our systematic review and meta-analysis results indicate that heparin and its derivatives have anti-cancer properties in preclinical murine models of human and murine cancer cell line origin. Pertinently, newly developed heparin derivatives also exhibited positive anti-cancer findings with little side effects. Future studies should focus on such new heparin derivatives, including LHT7, LHTD4, and non-anticoagulants compounds of heparin. In the same manner, warfarin exhibited anti-cancer effects in preclinical cancer models, while newer direct oral AC agents showed unconclusive results in our systematic review and meta-analysis.

Our findings highlight the need for future studies to optimize the use of anticoagulants (ACs) in cancer treatment within preclinical models, specifically by examining their interactions with chemotherapeutic agents to explore translational potential. The demonstrated anticancer properties of these compounds provide a strong basis for their evaluation in clinical settings, particularly newer heparin derivatives. If validated in human trials, these results could lead to the integration of ACs into cancer treatment regimens, especially in combination with chemotherapy, potentially enhancing therapeutic efficacy and influencing future treatment guidelines.

## Data Availability

The original contributions presented in the study are included in the article/Supplementary Material, further inquiries can be directed to the corresponding authors.

## References

[1] Ferlay J Colombet M Soerjomataram I Parkin DM Piñeros M Znaor A Cancer statistics for the year 2020: an overview. Int J Cancer. (2021) 149:778–89. 10.1002/ijc.33588 33818764

[2] Organization WH. WHO Global Survey on the Inclusion of Cancer Care in Health-Benefit Packages, 2020–2021. Geneva: World Health Organization (2024).

[3] Ma X Lakshmipriya T Gopinath SCB. Recent advances in identifying biomarkers and high-affinity aptamers for gynecologic cancers diagnosis and therapy. J Anal Methods Chem. (2019) 2019:5426974. 10.1155/2019/5426974 31583159 PMC6754908

[4] Lewandowska AM Rudzki M Rudzki S Lewandowski T Laskowska B. Environmental risk factors for cancer—review paper. Ann Agric Environ Med. (2019) 26(1):1–7. 10.26444/aaem/94299 30922021

[5] Debela DT Muzazu SG Heraro KD Ndalama MT Mesele BW Haile DC New approaches and procedures for cancer treatment: current perspectives. SAGE Open Med. (2021) 9:20503121211034366. 10.1177/20503121211034366 34408877 PMC8366192

[6] Paul S Konig MF Pardoll DM Bettegowda C Papadopoulos N Wright KM Cancer therapy with antibodies. Nat Rev Cancer. (2024) 24:399–426. 10.1038/s41568-024-00690-x 38740967 PMC11180426

[7] Hebert JD Neal JW Winslow MM. Dissecting metastasis using preclinical models and methods. Nat Rev Cancer. (2023) 23(6):391–407. 10.1038/s41568-023-00568-4 37138029

[8] Agnelli G Verso M. Management of venous thromboembolism in patients with cancer. J Thromb Haemost. (2011) 9(Suppl 1):316–24. 10.1111/j.1538-7836.2011.04346.x 21781268

[9] Khorana AA Mackman N Falanga A Pabinger I Noble S Ageno W Cancer-associated venous thromboembolism. Nat Rev Dis Primers. (2022) 8(1):11. 10.1038/s41572-022-00336-y 35177631

[10] Borsig L. Antimetastatic activities of heparins and modified heparins. Experimental evidence. Thromb Res. (2010) 125(Suppl 2):S66–71. 10.1016/S0049-3848(10)70017-7 20434009

[11] Najidh S Versteeg HH Buijs JT. A systematic review on the effects of direct oral anticoagulants on cancer growth and metastasis in animal models. Thromb Res. (2020) 187:18–27. 10.1016/j.thromres.2019.12.022 31945588

[12] Achen MG Mann GB Stacker SA. Targeting lymphangiogenesis to prevent tumour metastasis. Br J Cancer. (2006) 94(10):1355–60. 10.1038/sj.bjc.6603120 16641900 PMC2361285

[13] Thaler J Pabinger I Ay C. Anticoagulant treatment of deep vein thrombosis and pulmonary embolism: the present state of the art. Front Cardiovasc Med. (2015) 2:30. 10.3389/fcvm.2015.00030 26664901 PMC4671349

[14] Franco Moreno AI Martín Díaz RM García Navarro MJ. Anticoagulantes orales directos: puesta al día. Med Clin (Barc). (2018) 151(5):198–206. 10.1016/j.medcli.2017.11.042 29295790

[15] Kirane A Ludwig KF Sorrelle N Haaland G Sandal T Ranaweera R Warfarin blocks Gas6-mediated axl activation required for pancreatic cancer epithelial plasticity and metastasis. Cancer Res. (2015) 75(18):3699–705. 10.1158/0008-5472.CAN-14-2887-T 26206560 PMC4572915

[16] Wojtukiewicz MZ Skalij P Tokajuk P Politynska B Wojtukiewicz AM Tucker SC Direct oral anticoagulants in cancer patients. Time for a change in paradigm. Cancers (Basel). (2020) 12(5):1144. 10.3390/cancers12051144 32370207 PMC7281117

[17] Page MJ McKenzie JE Bossuyt PM Boutron I Hoffmann TC Mulrow CD The PRISMA 2020 statement: an updated guideline for reporting systematic reviews. Br Med J. (2021) 372:n71. 10.1136/bmj.n71 33782057 PMC8005924

[18] Boeryd B. Action of heparin and plasminogen inhibitor (EACA) on metastatic tumour spread in an isologous system. Acta Pathol Microbiol Scand. (1965) 65(3):395–404. 10.1111/apm.1965.65.3.395 5884701

[19] Boeryd B. Effect of heparin and plasminogen inhibitor (EACA) in brief and prolonged treatment on intravenously injected tumour cells. Acta Pathol Microbiol Scand. (1966) 68(3):347–54. 10.1111/apm.1966.68.3.347 5959842

[20] Boeryd B Rudenstam CM. Effect of heparin, plasminogen inhibitor (EACA) and trauma on tumour metastases. Acta Pathol Microbiol Scand. (1967) 69(1):28–34. 10.1111/j.1699-0463.1967.tb05123.x

[21] Hagmar B. Tumour growth and spontaneous metastasis spread in two syngeneic systems. Acta Pathol Microbiol Scand A. (1970) 78(2):131–42. 10.1111/j.1699-0463.1970.tb00248.x 5428276

[22] Hagmar B Norrby K. Evidence for effects of heparin on cell surfaces influencing experimental metastases. Int J Cancer. (1970) 5(1):72–84. 10.1002/ijc.2910050110 5414745

[23] Koike A. Mechanism of blood-borne metastases. I. Some factors affecting lodgment and growth of tumor cells in the lungs. Cancer. (1964) 17:450–60. 10.1002/1097-0142(196404)17:4<450::AID-CNCR2820170406>3.0.CO;2-2 14136528

[24] Monzavi-Karbassi B Stanley JS Hennings L Jousheghany F Artaud C Shaaf S Chondroitin sulfate glycosaminoglycans as major P-selectin ligands on metastatic breast cancer cell lines. Int J Cancer. (2007) 120(6):1179–91. 10.1002/ijc.22424 17154173

[25] Beuth J Ko HL Uhlenbruck G Pulverer G. Combined immunostimulation (Propionibacterium avidum KP 40) and anticoagulation (heparin) prevents metastatic lung and liver colonization in mice. J Cancer Res Clin Oncol. (1987) 113(4):359–62. 10.1007/BF00397719 3597521 PMC12248343

[26] Lee AE Rogers LA Longcroft JM Jeffery RE. Reduction of metastasis in a murine mammary tumour model by heparin and polyinosinic-polycytidylic acid. Clin Exp Metastasis. (1990) 8(2):165–71. 10.1007/BF00117789 2317956

[27] Lee JK Choi B Sobel RA Chiocca EA Martuza RL. Inhibition of growth and angiogenesis of human neurofibrosarcoma by heparin and hydrocortisone. J Neurosurg. (1990) 73(3):429–35. 10.3171/jns.1990.73.3.0429 1696626

[28] Lee AE Rogers LA Jeffery RE Longcroft JM. Comparison of metastatic cell lines derived from a murine mammary tumour, and reduction of metastasis by heparin. Clin Exp Metastasis. (1988) 6(6):463–71. 10.1007/BF01784377 3409560

[29] Lione A Bosmann HB. The inhibitory effect of heparin and warfarin treatments on the intravascular survival of B16 melanoma cells in syngeneic C57 mice. Cell Biol Int Rep. (1978) 2(1):81–6. 10.1016/0309-1651(78)90087-5 630615

[30] Murata J Saiki I Nishimura S Nishi N Tokura S Azuma I. Inhibitory effect of chitin heparinoids on the lung metastasis of B16-BL6 melanoma. Jpn J Cancer Res. (1989) 80(9):866–72. 10.1111/j.1349-7006.1989.tb01728.x 2513303 PMC5917858

[31] Rossi C Hess S Eckl RW di Lena A Bruno A Thomas O Effect of MCM09, an active site-directed inhibitor of factor Xa, on B16-BL6 melanoma lung colonies in mice. J Thromb Haemost. (2006) 4(3):608–13. 10.1111/j.1538-7836.2006.01793.x 16460443

[32] Szende B Paku S Rácz G Kopper L. Effect of fraxiparine and heparin on experimental tumor metastasis in mice. Anticancer Res. (2005) 25(4):2869–72. 16080539

[33] Back N Leblanc PP. Proteases during the growth of ehrlich ascites tumor. III. Effect of e-aminocaproic acid (EACA) and heparin. Eur J Cancer (1965). (1977) 13(9):947–50. 10.1016/0014-2964(77)90170-0 913477

[34] Hagmar B. Cell surface charge and metastasis formation. A study on the effects of dextrans and heparin on tumour cells and experimental metastases in a syngeneic murine system. Acta Pathol Microbiol Scand A. (1972) 80(3):357–66. 5045416

[35] Ichikawa J Cole HA Magnussen RA Mignemi NA Butler M Holt GE Thrombin induces osteosarcoma growth, a function inhibited by low molecular weight heparin *in vitro* and *in vivo*: procoagulant nature of osteosarcoma. Cancer. (2012) 118(9):2494–506. 10.1002/cncr.26518 21953059

[36] Murata J Saiki I Makabe T Tsuta Y Tokura S Azuma I. Inhibition of tumor-induced angiogenesis by sulfated chitin derivatives. Cancer Res. (1991) 51(1):22–6. 1703032

[37] Colucci M Delaini F de Bellis Vitti G Locati D Poggi A Semeraro N Warfarin inhibits both procoagulant activity and metastatic capacity of Lewis lung carcinoma cells. Biochem Pharmacol. (1983) 32(11):1689–91. 10.1016/0006-2952(83)90110-7 6870908

[38] Gorelik E. Protective effect of fibrin on tumour metastasis. Fibrinolysis. (1992) 6:35–8. 10.1016/0268-9499(92)90091-U

[39] Borsig L Wong R Hynes RO Varki NM Varki A. Synergistic effects of L- and P-selectin in facilitating tumor metastasis can involve non-mucin ligands and implicate leukocytes as enhancers of metastasis. Proc Natl Acad Sci U S A. (2002) 99(4):2193–8. 10.1073/pnas.261704098 11854515 PMC122341

[40] Poggi A Rossi C Casella N Bruno C Sturiale L Dossi C Inhibition of B16-BL6 melanoma lung colonies by semisynthetic sulfaminoheparosan sulfates from E. coli K5 polysaccharide. Semin Thromb Hemost. (2002) 28(4):383–92. 10.1055/s-2002-34308 12244486

[41] Nicolson GL Nakajima M Wakabayashi H Boyd DD Diaz D Irimura T. Cancer cell heparanase activity associated with invasion and metastasis. Adv Enzyme Regul. (1998) 38:19–32. 10.1016/S0065-2571(97)00016-2 9762344

[42] Sudha T Yalcin M Lin HY Elmetwally AM Nazeer T Arumugam T Suppression of pancreatic cancer by sulfated non-anticoagulant low molecular weight heparin. Cancer Lett. (2014) 350(1-2):25–33. 10.1016/j.canlet.2014.04.016 24769074 PMC4066671

[43] Gorelik E. Augmentation of the antimetastatic effect of anticoagulant drugs by immunostimulation in mice. Cancer Res. (1987) 47(3):809–15. 3802083

[44] Alonso D Bertolesi G Farias E Eijan A Joffe E Decidre L. Antimetastatic effects associated with anticoagulant properties of heparin and chemically modified heparin species in a mouse mammary tumor model. Oncol Rep. (1996) 3(1):219–22. 10.3892/or.3.1.219 21594348

[45] Bereczky B Gilly R Rásó E Vágó A Tímár J Tóvári J. Selective antimetastatic effect of heparins in preclinical human melanoma models is based on inhibition of migration and microvascular arrest. Clin Exp Metastasis. (2005) 22(1):69–76. 10.1007/s10585-005-3859-6 16132580

[46] Bobek V Boubelik M Fiserová A L'Uptovcová M Vannucci L Kacprzak G Anticoagulant drugs increase natural killer cell activity in lung cancer. Lung Cancer. (2005) 47(2):215–23. 10.1016/j.lungcan.2004.06.012 15639720

[47] Boeryd B Hagmar B. Disappearance of circulating tumour cells in mice treated with heparin, coumarin and EACA. Acta Pathol Microbiol Scand A. (1972) 80(3):303–7. 10.1111/j.1699-0463.1972.tb00284.x 5045410

[48] Borsig L Wong R Feramisco J Nadeau DR Varki NM Varki A. Heparin and cancer revisited: mechanistic connections involving platelets, P-selectin, carcinoma mucins, and tumor metastasis. Proc Natl Acad Sci U S A. (2001) 98(6):3352–7. 10.1073/pnas.061615598 11248082 PMC30657

[49] Brito AS Cavalcante RS Cavalheiro RP Palhares L Nobre L Andrade GPV Anti-IIa activity and antitumor properties of a hybrid heparin/heparan sulfate-like compound from litopenaeus vannamei shrimp. Int J Biol Macromol. (2018) 118(Pt B):1470–8. 10.1016/j.ijbiomac.2018.06.143 29964117

[50] el Rifi K Bacon B Mehigan J Hoppe E Cole WH. Increased incidence of pulmonary metastases after celiotomy: counteraction by heparin. Arch Surg. (1965) 91(4):625–9. 10.1001/archsurg.1965.01320160079018 5836114

[51] Goldie H Walker M Biscoe B Powell GJ Howse RJ. Growth characteristics of ascitic tumor cells in the heparinized peritoneal cavity of the mouse. Proc Soc Exp Biol Med. (1961) 107:838–42. 10.3181/00379727-107-26771 13899773

[52] Goldstein DS Lu ML Hattori T Ratliff TL Loughlin KR Kavoussi LR. Inhibition of peritoneal tumor-cell implantation: model for laparoscopic cancer surgery. J Endourol. (1993) 7(3):237–41. 10.1089/end.1993.7.237 8358421

[53] Gorelik E Bere WW Herberman RB. Role of NK cells in the antimetastatic effect of anticoagulant drugs. Int J Cancer. (1984) 33(1):87–94. 10.1002/ijc.2910330115 6363308

[54] Harvey JR Mellor P Eldaly H Lennard TW Kirby JA Ali S. Inhibition of CXCR4-mediated breast cancer metastasis: a potential role for heparinoids? Clin Cancer Res. (2007) 13(5):1562–70. 10.1158/1078-0432.CCR-06-1987 17332302

[55] Irimura T Nakajima M Nicolson GL. Chemically modified heparins as inhibitors of heparan sulfate specific endo-.beta.-glucuronidase (heparanase) of metastatic melanoma cells. Biochemistry. (1986) 25(18):5322–8. 10.1021/bi00366a050 3768351

[56] Kreisler L. Effect of heparin on the growth of a transplantable lymphosarcoma in mice. Science. (1952) 115(2980):145–6. 10.1126/science.115.2980.145 14913196

[57] Lippman M. The growth-inhibitory action of heparin on the ehrlich ascites tumor in mice. Cancer Res. (1957) 17(1):11–4. 13413829

[58] Ludwig RJ Alban S Bistrian R Boehncke WH Kaufmann R Henschler R The ability of different forms of heparins to suppress P-selectin function *in vitro* correlates to their inhibitory capacity on bloodborne metastasis *in vivo*. Thromb Haemost. (2006) 95(3):535–40. 10.1160/TH05-07-0515 16525583

[59] Ludwig RJ Boehme B Podda M Henschler R Jager E Tandi C Endothelial P-selectin as a target of heparin action in experimental melanoma lung metastasis. Cancer Res. (2004) 64(8):2743–50. 10.1158/0008-5472.CAN-03-1054 15087389

[60] Ma L Qiao H He C Yang Q Cheung CH Kanwar JR Modulating the interaction of CXCR4 and CXCL12 by low-molecular-weight heparin inhibits hepatic metastasis of colon cancer. Invest New Drugs. (2012) 30(2):508–17. 10.1007/s10637-010-9578-0 21080209

[61] Maat B. Extrapulmonary colony formation after intravenous injection of tumour cells into heparin-treated animals. Br J Cancer. (1978) 37(3):369–76. 10.1038/bjc.1978.56 638015 PMC2009537

[62] Maat B Hilgard P. Anticoagulants and experimental metastases-evaluation of antimetastatic effects in different model systems. J Cancer Res Clin Oncol. (1981) 101(3):275–83. 10.1007/BF00410113 7309780 PMC12253567

[63] Mellor P Harvey JR Murphy KJ Pye D O'Boyle G Lennard TW Modulatory effects of heparin and short-length oligosaccharides of heparin on the metastasis and growth of LMD MDA-MB 231 breast cancer cells *in vivo*. Br J Cancer. (2007) 97(6):761–8. 10.1038/sj.bjc.6603928 17726466 PMC2360379

[64] Milas L Hunter N Basic I. Treatment with cortisone plus heparin or hexuronyl hexoaminoglycan sulfates of murine tumors and their lung deposits. Clin Exp Metastasis. (1985) 3(4):247–55. 10.1007/BF01585080 4075612

[65] Murata J Saiki I Matsuno K Tokura S Azuma I. Inhibition of tumor cell arrest in lungs by antimetastatic chitin heparinoid. Jpn J Cancer Res. (1990) 81(5):506–13. 10.1111/j.1349-7006.1990.tb02599.x 2116400 PMC5918061

[66] Murthy MS Summaria LJ Miller RJ Wyse TB Goldschmidt RA Scanlon EF. Inhibition of tumor implantation at sites of trauma by plasminogen activators. Cancer. (1991) 68(8):1724–30.<1724::AID-CNCR2820680813>3.0.CO;2-W 1913515 10.1002/1097-0142(19911015)68:8<1724::aid-cncr2820680813>3.0.co;2-w

[67] Pan Y Zhong LJ Zhou H Wang X Chen K Yang HP Roles of vimentin and 14-3-3 zeta/delta in the inhibitory effects of heparin on PC-3M cell proliferation and B16-F10-luc-G5 cells metastasis. Acta Pharmacol Sin. (2012) 33(6):798–808. 10.1038/aps.2012.42 22669117 PMC4010382

[68] Park K Ki Lee S Hyun Son D Ah Park S Kim K Won Chang H The attenuation of experimental lung metastasis by a bile acid acylated-heparin derivative. Biomaterials. (2007) 28(16):2667–76. 10.1016/j.biomaterials.2007.02.001 17335894

[69] Park K Kim YS Lee GY Nam JO Lee SK Park RW Antiangiogenic effect of bile acid acylated heparin derivative. Pharm Res. (2006) 24(1):176–85. 10.1007/s11095-006-9139-6 17109210

[70] Park K Lee GY Park RW Kim IS Kim SY Byun Y. Combination therapy of heparin-deoxycholic acid conjugate and doxorubicin against squamous cell carcinoma and B16F10 melanoma. Pharm Res. (2008) 25(2):268–76. 10.1007/s11095-007-9366-5 17619999

[71] Sento S Sasabe E Yamamoto T. Application of a persistent heparin treatment inhibits the malignant potential of oral squamous carcinoma cells induced by tumor cell-derived exosomes. PLoS One. (2016) 11(2):e0148454. 10.1371/journal.pone.0148454 26849680 PMC4743844

[72] Shen X Fang J Lv X Pei Z Wang Y Jiang S Heparin impairs angiogenesis through inhibition of microRNA-10b. J Biol Chem. (2011) 286(30):26616–27. 10.1074/jbc.M111.224212 21642433 PMC3143626

[73] Stevenson JL Choi SH Varki A. Differential metastasis inhibition by clinically relevant levels of heparins—correlation with selectin inhibition, not antithrombotic activity. Clin Cancer Res. (2005) 11(19 Pt 1):7003–11. 10.1158/1078-0432.CCR-05-1131 16203794

[74] Stevenson JL Varki A Borsig L. Heparin attenuates metastasis mainly due to inhibition of P- and L-selectin, but non-anticoagulant heparins can have additional effects. Thromb Res. (2007) 120(Suppl 2):S107–11. 10.1016/S0049-3848(07)70138-X 18023703

[75] Sylvester DM Liu SY Meadows GG. Augmentation of antimetastatic activity of interferon and tumor necrosis factor by heparin. Immunopharmacol Immunotoxicol. (1990) 12(2):161–80. 10.3109/08923979009019667 2121817

[76] Takahashi H Ebihara S Okazaki T Asada M Sasaki H Yamaya M. A comparison of the effects of unfractionated heparin, dalteparin and danaparoid on vascular endothelial growth factor-induced tumour angiogenesis and heparanase activity. Br J Pharmacol. (2005) 146(3):333–43. 10.1038/sj.bjp.0706344 16041398 PMC1576289

[77] Thurston G Smith KA Murray JC. Anticoagulant treatment does not affect the action of flavone acetic acid in tumour-bearing mice. Br J Cancer. (1991) 64(4):689–92. 10.1038/bjc.1991.382 1911218 PMC1977683

[78] Wei W Zuo Y Hu Y Wang L Jia L Zhang J. Heparin inhibits P388D1 cells adherence and metastasis to peripheral lymph nodes *in vitro* and *in vivo*. Lymphology. (2009) 42(1):10–8. 19499763

[79] Wheatley DN. Influence of various substances on production of ascites tumour. Nature. (1964) 202:1348–9. 10.1038/2021348a0 14210984

[80] Yee CK Butcher M Zeadin M Weitz JI Shaughnessy SG. Inhibition of osteolytic bone metastasis by unfractionated heparin. Clin Exp Metastasis. (2008) 25(8):903–11. 10.1007/s10585-008-9212-0 18814041

[81] Yin W Zhang J Jiang Y Juan S. Combination therapy with low molecular weight heparin and Adriamycin results in decreased breast cancer cell metastasis in C3H mice. Exp Ther Med. (2014) 8(4):1213–8. 10.3892/etm.2014.1911 25187827 PMC4151689

[82] Yoshitomi Y Nakanishi H Kusano Y Munesue S Oguri K Tatematsu M Inhibition of experimental lung metastases of Lewis lung carcinoma cells by chemically modified heparin with reduced anticoagulant activity. Cancer Lett. (2004) 207(2):165–74. 10.1016/j.canlet.2003.11.037 15072825

[83] Yu L Garg HG Li B Linhardt RJ Hales CA. Antitumor effect of butanoylated heparin with low anticoagulant activity on lung cancer growth in mice and rats. Curr Cancer Drug Targets. (2010) 10(2):229–41. 10.2174/156800910791054176 20201787

[84] Zhang C Liu Y Gao Y Shen J Zheng S Wei M Modified heparins inhibit integrin alpha(IIb)beta(3) mediated adhesion of melanoma cells to platelets *in vitro* and *in vivo*. Int J Cancer. (2009) 125(9):2058–65. 10.1002/ijc.24561 19598263

[85] Lee DY Park K Kim SK Park RW Kwon IC Kim SY Antimetastatic effect of an orally active heparin derivative on experimentally induced metastasis. Clin Cancer Res. (2008) 14(9):2841–9. 10.1158/1078-0432.CCR-07-0641 18451252

[86] Cheng W Dahmani FZ Zhang J Xiong H Wu Y Yin L Anti-angiogenic activity and antitumor efficacy of amphiphilic twin drug from ursolic acid and low molecular weight heparin. Nanotechnology. (2017) 28(7):075102. 10.1088/1361-6528/aa53c6 28091396

[87] Luengo-Gil G Calvo MI Martín-Villar E Águila S Bohdan N Antón AI Antithrombin controls tumor migration, invasion and angiogenesis by inhibition of enteropeptidase. Sci Rep. (2016) 6:27544. 10.1038/srep27544 27270881 PMC4897635

[88] Niu Q Wang W Li Y Ruden DM Wang F Li Y Low molecular weight heparin ablates lung cancer cisplatin-resistance by inducing proteasome-mediated ABCG2 protein degradation. PLoS One. (2012) 7(7):e41035. 10.1371/journal.pone.0041035 22844424 PMC3402471

[89] Park J Kim JY Hwang SR Mahmud F Byun Y. Chemical conjugate of low molecular weight heparin and suramin fragment inhibits tumor growth possibly by blocking VEGF165. Mol Pharm. (2015) 12(11):3935–42. 10.1021/acs.molpharmaceut.5b00348 26448404

[90] Park JW Jeon OC Kim SK Al-Hilal TA Jin SJ Moon HT High antiangiogenic and low anticoagulant efficacy of orally active low molecular weight heparin derivatives. J Control Release. (2010) 148(3):317–26. 10.1016/j.jconrel.2010.09.014 20869408

[91] Takeuchi A Yamamoto Y Munesue S Harashima A Watanabe T Yonekura H Low molecular weight heparin suppresses receptor for advanced glycation end products-mediated expression of malignant phenotype in human fibrosarcoma cells. Cancer Sci. (2013) 104(6):740–9. 10.1111/cas.12133 23421467 PMC7657155

[92] Zhou Z Peng Y Ai W Li Q Ye T Wu C Compound opening arrow mixture exerts anti-tumor effects in a mouse model of breast cancer. Sci Rep. (2020) 10(1):8175. 10.1038/s41598-020-64561-9 32424152 PMC7235040

[93] Santos RP Tovar AMF Oliveira MR Piquet AA Capillé NV Oliveira S Pharmacokinetic, hemostatic, and anticancer properties of a low-anticoagulant bovine heparin. TH Open. (2022) 6(2):e114–e23. 10.1055/s-0042-1745743 35707626 PMC9135479

[94] Amirkhosravi A Mousa SA Amaya M Francis JL. Antimetastatic effect of tinzaparin, a low-molecular-weight heparin. J Thromb Haemost. (2003) 1(9):1972–6. 10.1046/j.1538-7836.2003.00341.x 12941039

[95] Phillips PG Yalcin M Cui H Abdel-Nabi H Sajjad M Bernacki R Increased tumor uptake of chemotherapeutics and improved chemoresponse by novel non-anticoagulant low molecular weight heparin. Anticancer Res. (2011) 31(2):411–9. 21378319

[96] Alyahya R Sudha T Racz M Stain SC Mousa SA. Anti-metastasis efficacy and safety of non-anticoagulant heparin derivative versus low molecular weight heparin in surgical pancreatic cancer models. Int J Oncol. (2015) 46(3):1225–31. 10.3892/ijo.2014.2803 25530018

[97] Seth R Tai LH Falls T de Souza CT Bell JC Carrier M Surgical stress promotes the development of cancer metastases by a coagulation-dependent mechanism involving natural killer cells in a murine model. Ann Surg. (2013) 258(1):158–68. 10.1097/SLA.0b013e31826fcbdb 23108132

[98] Feinauer MJ Schneider SW Berghoff AS Robador JR Tehranian C Karreman MA Local blood coagulation drives cancer cell arrest and brain metastasis in a mouse model. Blood. (2021) 137(9):1219–32. 10.1182/blood.2020005710 33270819

[99] Bauer AT Suckau J Frank K Desch A Goertz L Wagner AH von Willebrand factor fibers promote cancer-associated platelet aggregation in malignant melanoma of mice and humans. Blood. (2015) 125(20):3153–63. 10.1182/blood-2014-08-595686 25977583 PMC4432010

[100] Goertz L Schneider SW Desch A Mayer FT Koett J Nowak K Heparins that block VEGF-A-mediated von willebrand factor fiber generation are potent inhibitors of hematogenous but not lymphatic metastasis. Oncotarget. (2016) 7(42):68527–45. 10.18632/oncotarget.11832 27602496 PMC5356571

[101] Kragh M Binderup L Vig Hjarnaa PJ Bramm E Johansen KB Frimundt Petersen C. Non-anti-coagulant heparin inhibits metastasis but not primary tumor growth. Oncol Rep. (2005) 14(1):99–104. 15944775

[102] Mueller T Pfankuchen DB Wantoch von Rekowski K Schlesinger M Reipsch F Bendas G. The impact of the low molecular weight heparin tinzaparin on the sensitization of cisplatin-resistant ovarian cancers-preclinical *in vivo* evaluation in Xenograft tumor models. Molecules. (2017) 22(5):728. 10.3390/molecules22050728 28467373 PMC6154624

[103] Sarantis P Bokas A Papadimitropoulou A Koustas E Theocharis S Papakotoulas P Combinatorial treatment of tinzaparin and chemotherapy can induce a significant antitumor effect in pancreatic cancer. Int J Mol Sci. (2021) 22(13):7053. 10.3390/ijms22137053 34208987 PMC8268558

[104] Schlesinger M Roblek M Ortmann K Naggi A Torri G Borsig L The role of VLA-4 binding for experimental melanoma metastasis and its inhibition by heparin. Thromb Res. (2014) 133(5):855–62. 10.1016/j.thromres.2014.02.020 24636486

[105] Rui Y Wang D Hu D Huang L. Role of dalteparin sodium on the growth of cancer cells and tumor-associated angiogenesis in A549 human lung cancer cell line and grafted mouse model. J Cancer Res Ther. (2018) 14(Supplement):S985–s92. 10.4103/0973-1482.192765 30539834

[106] Buijs JT Laghmani EH van den Akker RFP Tieken C Vletter EM van der Molen KM The direct oral anticoagulants rivaroxaban and dabigatran do not inhibit orthotopic growth and metastasis of human breast cancer in mice. J Thromb Haemost. (2019) 17(6):951–63. 10.1111/jth.14443 30929299 PMC6849835

[107] Graf C Wilgenbus P Pagel S Pott J Marini F Reyda S Myeloid cell-synthesized coagulation factor X dampens antitumor immunity. Sci Immunol. (2019) 4(39):eaaw8405. 10.1126/sciimmunol.aaw8405 31541031 PMC6830514

[108] Lund EL Olsen MW Lipson KE McMahon G Howlett AR Kristjansen PE. Improved effect of an antiangiogenic tyrosine kinase inhibitor (SU5416) by combinations with fractionated radiotherapy or low molecular weight heparin. Neoplasia. (2003) 5(2):155–60. 10.1016/S1476-5586(03)80007-6 12659688 PMC1502401

[109] Klerk CP Niers TM Brüggemann LW Smorenburg SM Richel DJ Spek CA Prophylactic plasma levels of the low molecular weight heparin nadroparin does not affect colon cancer tumor development in mouse liver. Thromb Res. (2010) 125(3):235–8. 10.1016/j.thromres.2009.03.005 19371924

[110] Wasiutynski A Skopinska-Rozewska E Jung L Sommer E Bany J Siwicki A Comparison of the effects of enoxaparin and nadroparin on tumor angiogenesis in mice. Cent Eur J Immunol. (2006) 31(1/2):70.

[111] Zhuang X Qiao T Yuan S Zhang Q Chen W Luo Y Antitumor effects of nadroparin combined with radiotherapy in Lewis lung cancer models. Onco Targets Ther. (2018) 11:5133–42. 10.2147/OTT.S176526 30210234 PMC6114476

[112] Niers TM Brüggemann LW Klerk CP Muller FJ Buckle T Reitsma PH Differential effects of anticoagulants on tumor development of mouse cancer cell lines B16, K1735 and CT26 in lung. Clin Exp Metastasis. (2009) 26(3):171–8. 10.1007/s10585-008-9227-6 19067186

[113] Skopińska-Różewska E Skurzak H Wasiutyński A Jung L Siwicki AK Bałan BJ Experimental immunology sarcoma L-1 in mice as a model for the study of experimental angiogenesis. Cent Eur J Immunol. (2007) 32(2):77–83.

[114] Mousa SA Linhardt R Francis JL Amirkhosravi A. Anti-metastatic effect of a non-anticoagulant low-molecular-weight heparin versus the standard low-molecular-weight heparin, enoxaparin. Thromb Haemost. (2006) 96(6):816–21. 10.1160/th06-05-0289 17139378 PMC4114246

[115] Djaafar S Dunand-Sautier I Gonelle-Gispert C Lacotte S Agostini AD Petro M Enoxaparin attenuates mouse colon cancer liver metastases by inhibiting heparanase and interferon-*γ*-inducible chemokines. Anticancer Res. (2016) 36(8):4019–32. 27466508

[116] Stocking KL Jones JC Everds NE Buetow BS Roudier MP Miller RE. Use of low-molecular-weight heparin to decrease mortality in mice after intracardiac injection of tumor cells. Comp Med. (2009) 59(1):37–45. 19295053 PMC2703139

[117] Van Sluis GL Nieuwdorp M Kamphuisen PW van der Vlag J Van Noorden CJ Spek CA. A low molecular weight heparin inhibits experimental metastasis in mice independently of the endothelial glycocalyx. PLoS One. (2010) 5(6):e11200. 10.1371/journal.pone.0011200 20574516 PMC2888573

[118] Pollari S Käkönen RS Mohammad KS Rissanen JP Halleen JM Wärri A Heparin-like polysaccharides reduce osteolytic bone destruction and tumor growth in a mouse model of breast cancer bone metastasis. Mol Cancer Res. (2012) 10(5):597–604. 10.1158/1541-7786.MCR-11-0482 22522458

[119] MacDonald A Priess M Curran J Guess J Farutin V Oosterom I Necuparanib, a multitargeting heparan sulfate mimetic, targets tumor and stromal compartments in pancreatic cancer. Mol Cancer Ther. (2019) 18(2):245–56. 10.1158/1535-7163.MCT-18-0417 30401693

[120] Lee GY Kim SK Byun Y. Glucosylated heparin derivatives as non-toxic anti-cancer drugs. J Control Release. (2007) 123(1):46–55. 10.1016/j.jconrel.2007.07.017 17765351

[121] Cassinelli G Dal Bo L Favini E Cominetti D Pozzi S Tortoreto M Supersulfated low-molecular weight heparin synergizes with IGF1R/IR inhibitor to suppress synovial sarcoma growth and metastases. Cancer Lett. (2018) 415:187–97. 10.1016/j.canlet.2017.12.009 29225052

[122] Lee E Kim YS Bae SM Kim SK Jin S Chung SW Polyproline-type helical-structured low-molecular weight heparin (LMWH)-taurocholate conjugate as a new angiogenesis inhibitor. Int J Cancer. (2009) 124(12):2755–65. 10.1002/ijc.24239 19243020

[123] Chung SW Bae SM Lee M Al-Hilal TA Lee CK Kim JK LHT7, a chemically modified heparin, inhibits multiple stages of angiogenesis by blocking VEGF, FGF2 and PDGF-B signaling pathways. Biomaterials. (2015) 37:271–8. 10.1016/j.biomaterials.2014.10.004 25453957

[124] Kim JY Chung SW Kim SY Byun Y. Enhanced anti-angiogenic effect of low molecular weight heparin-bile acid conjugates by co-administration of a selective COX-2 inhibitor. Pharm Res. (2015) 32(7):2318–27. 10.1007/s11095-015-1623-4 25585956

[125] Adulnirath A Chung SW Park J Hwang SR Kim JY Yang VC Cyclic RGDyk-conjugated LMWH-taurocholate derivative as a targeting angiogenesis inhibitor. J Control Release. (2012) 164(1):8–16. 10.1016/j.jconrel.2012.10.001 23063549

[126] Alam F Hwang SR Al-Hilal TA Chung SW Kim HS Kang BH Safety studies on intravenous infusion of a potent angiogenesis inhibitor: taurocholate-conjugated low molecular weight heparin derivative LHT7 in preclinical models. Drug Dev Ind Pharm. (2016) 42(8):1247–57. 10.3109/03639045.2015.1122609 26612099

[127] Alam F Al-Hilal TA Chung SW Seo D Mahmud F Kim HS Oral delivery of a potent anti-angiogenic heparin conjugate by chemical conjugation and physical complexation using deoxycholic acid. Biomaterials. (2014) 35(24):6543–52. 10.1016/j.biomaterials.2014.04.050 24816287

[128] Bae SM Kim JH Chung SW Byun Y Kim SY Lee BH An apoptosis-homing peptide-conjugated low molecular weight heparin-taurocholate conjugate with antitumor properties. Biomaterials. (2013) 34(8):2077–86. 10.1016/j.biomaterials.2012.11.020 23245333

[129] Alam F Al-Hilal TA Park J Choi JU Mahmud F Jeong JH Multi-stage inhibition in breast cancer metastasis by orally active triple conjugate, LHTD4 (low molecular weight heparin-taurocholate-tetrameric deoxycholate). Biomaterials. (2016) 86:56–67. 10.1016/j.biomaterials.2016.01.058 26890038

[130] Kim JY Al-Hilal TA Chung SW Kim SY Ryu GH Son WC Antiangiogenic and anticancer effect of an orally active low molecular weight heparin conjugates and its application to lung cancer chemoprevention. J Control Release. (2015) 199:122–31. 10.1016/j.jconrel.2014.12.015 25523032

[131] Choi JU Chung SW Al-Hilal TA Alam F Park J Mahmud F A heparin conjugate, LHbisD4, inhibits lymphangiogenesis and attenuates lymph node metastasis by blocking VEGF-C signaling pathway. Biomaterials. (2017) 139:56–66. 10.1016/j.biomaterials.2017.05.026 28586719

[132] Cho KJ Moon HT Park GE Jeon OC Byun Y Lee YK. Preparation of sodium deoxycholate (DOC) conjugated heparin derivatives for inhibition of angiogenesis and cancer cell growth. Bioconjug Chem. (2008) 19(7):1346–51. 10.1021/bc800173m 18588324

[133] Lee DY Kim SK Kim YS Son DH Nam JH Kim IS Suppression of angiogenesis and tumor growth by orally active deoxycholic acid-heparin conjugate. J Control Release. (2007) 118(3):310–7. 10.1016/j.jconrel.2006.12.031 17291620

[134] Lee DY Lee SW Kim SK Lee M Chang HW Moon HT Antiangiogenic activity of orally absorbable heparin derivative in different types of cancer cells. Pharm Res. (2009) 26(12):2667–76. 10.1007/s11095-009-9989-9 19830530

[135] Park J Jeong JH Al-Hilal TA Kim JY Byun Y. Size controlled heparin fragment–deoxycholic acid conjugate showed anticancer property by inhibiting VEGF_165_. Bioconjug Chem. (2015) 26(5):932–40. 10.1021/acs.bioconjchem.5b00133 25894217

[136] Yu MK Lee DY Kim YS Park K Park SA Son DH Antiangiogenic and apoptotic properties of a novel amphiphilic folate-heparin-lithocholate derivative having cellular internality for cancer therapy. Pharm Res. (2007) 24(4):705–14. 10.1007/s11095-006-9190-3 17318418

[137] Sudha T Phillips P Kanaan C Linhardt RJ Borsig L Mousa SA. Inhibitory effect of non-anticoagulant heparin (S-NACH) on pancreatic cancer cell adhesion and metastasis in human umbilical cord vessel segment and in mouse model. Clin Exp Metastasis. (2012) 29(5):431–9. 10.1007/s10585-012-9461-9 22415710

[138] Ono K Ishihara M Ishikawa K Ozeki Y Deguchi H Sato M Periodate-treated, non-anticoagulant heparin-carrying polystyrene (NAC-HCPS) affects angiogenesis and inhibits subcutaneous induced tumour growth and metastasis to the lung. Br J Cancer. (2002) 86(11):1803–12. 10.1038/sj.bjc.6600307 12087470 PMC2375397

[139] Thorpe PE Derbyshire EJ Andrade SP Press N Knowles PP King S Heparin-steroid conjugates: new angiogenesis inhibitors with antitumor activity in mice. Cancer Res. (1993) 53(13):3000–7. 7686447

[140] Ritchie JP Ramani VC Ren Y Naggi A Torri G Casu B SST0001, a chemically modified heparin, inhibits myeloma growth and angiogenesis via disruption of the heparanase/syndecan-1 axis. Clin Cancer Res. (2011) 17(6):1382–93. 10.1158/1078-0432.CCR-10-2476 21257720 PMC3060291

[141] Cassinelli G Favini E Dal Bo L Tortoreto M De Maglie M Dagrada G Antitumor efficacy of the heparan sulfate mimic roneparstat (SST0001) against sarcoma models involves multi-target inhibition of receptor tyrosine kinases. Oncotarget. (2016) 7(30):47848–63. 10.18632/oncotarget.10292 27374103 PMC5216983

[142] Esposito E Vlodavsky I Barash U Roscilli G Milazzo FM Giannini G Novel N-acetyl-glycol-split heparin biotin-conjugates endowed with anti-heparanase activity. Eur J Med Chem. (2020) 186:111831. 10.1016/j.ejmech.2019.111831 31740052

[143] Hostettler N Naggi A Torri G Ishai-Michaeli R Casu B Vlodavsky I P-selectin- and heparanase-dependent antimetastatic activity of non-anticoagulant heparins. Faseb j. (2007) 21(13):3562–72. 10.1096/fj.07-8450com 17557930

[144] Roy S Lai H Zouaoui R Duffner J Zhou H Jayaraman LP Bioactivity screening of partially desulfated low-molecular-weight heparins: a structure/activity relationship study. Glycobiology. (2011) 21(9):1194–205. 10.1093/glycob/cwr053 21515908

[145] Lapierre F Holme K Lam L Tressler RJ Storm N Wee J Chemical modifications of heparin that diminish its anticoagulant but preserve its heparanase-inhibitory, angiostatic, anti-tumor and anti-metastatic properties. Glycobiology. (1996) 6(3):355–66. 10.1093/glycob/6.3.355 8724143

[146] Chen JL Hong J Lu JL Chen MX Chen WX Zhu JS Effect of non-anticoagulant N-desulfated heparin on expression of vascular endothelial growth factor, angiogenesis and metastasis of orthotopic implantation of human gastric carcinoma. World J Gastroenterol. (2007) 13(3):457–61. 10.3748/wjg.v13.i3.457 17230619 PMC4065905

[147] Baker D Elkon D Lim ML Constable W Rinehart L Wanebo H. The influence of warfarin of levamisole on the incidence of metastases following local irradiation of a solid tumor. Cancer. (1982) 49(3):427–33. 10.1002/1097-0142(19820201)49:3<427::AID-CNCR2820490306>3.0.CO;2-6 7059906

[148] Biggerstaff J Amirkhosravi A Francis JL. Three-dimensional visualization and quantitation of fibrin in solid tumors by confocal laser scanning microscopy. Cytometry. (1997) 29(2):122–7. 10.1002/(SICI)1097-0320(19971001)29:2<122::AID-CYTO4>3.0.CO;2-F 9332818

[149] Brown JM. A study of the mechanism by which anticoagulation with warfarin inhibits blood-borne metastases. Cancer Res. (1973) 33(6):1217–24. 4718672

[150] Carmel RJ Brown JM. The effect of cyclophosphamide and other drugs on the incidence of pulmonary metastases in mice. Cancer Res. (1977) 37(1):145–51. 137074

[151] Dumont P Atassi G De Jager R. Ineffectiveness of inicarone, a fibrinolytic agent, alone or in combination with chemotherapeutic agents on spontaneously metastasizing murine tumours. Clin Exp Metastasis. (1983) 1(4):349–57. 10.1007/BF00121197 6546206

[152] Fasco MJ Wilson AC Lincoln D Gierthy J. Evidence for a warfarin-sensitive serum factor that participates in factor x activation by Lewis lung tumor cells. Int J Cancer. (1987) 39(5):631–7. 10.1002/ijc.2910390515 3570555

[153] Ghersa P Donelli MG. Distribution and antitumoral activity of Adriamycin combined with warfarin in mice. Cancer Chemother Pharmacol. (1980) 5(1):43–7. 10.1007/BF00578561 7460193

[154] Ketcham AS Sugarbaker EV Ryan JJ Orme SK. Clotting factors and metastasis formation. Am J Roentgenol Radium Ther Nucl Med. (1971) 111(1):42–7. 10.2214/ajr.111.1.42 5540931

[155] Ocal O Pashkov V Kollipara RK Zolghadri Y Cruz VH Hale MA A rapid *in vivo* screen for pancreatic ductal adenocarcinoma therapeutics. Dis Model Mech. (2015) 8(10):1201–11. 10.1242/dmm.020933 26438693 PMC4610235

[156] Lorenzet R Bottazzi B Locati D Colucci M Mantovani A Semeraro N Failure to warfarin to affect the tissue factor activity and the metastatic potential of murine fibrosarcoma cells. Eur J Cancer Clin Oncol. (1985) 21(2):263–5. 10.1016/0277-5379(85)90182-8 3987761

[157] Maeda M Murakami M Takegami T Ota T. Promotion or suppression of experimental metastasis of B16 melanoma cells after oral administration of lapachol. Toxicol Appl Pharmacol. (2008) 229(2):232–8. 10.1016/j.taap.2008.01.008 18294668

[158] Ryan JJ Ketcham AS Wexler H. Warfarin treatment of mice bearing autochthonous tumors: effect on spontaneous metastases. Science. (1968) 162(3861):1493–4. 10.1126/science.162.3861.1493 5700070

[159] Ryan JJ Ketcham AS Wexler H. Reduced incidence of spontaneous metastases with long-term coumadin* therapy. Ann Surg. (1968) 168(1):163–8. 10.1097/00000658-196807000-00021 5673195 PMC1387203

[160] Ryan JJ Ketcham AS Wexler H. Warfarin therapy as an adjunct to the surgical treatment of malignant tumors in mice. Cancer Res. (1969) 29(12):2191–4. 5387259

[161] Alexander ET Minton AR Hayes CS Goss A Van Ryn J Gilmour SK. Thrombin inhibition and cyclophosphamide synergistically block tumor progression and metastasis. Cancer Biol Ther. (2015) 16(12):1802–11. 10.1080/15384047.2015.1078025 26383051 PMC4847815

[162] Alexander ET Minton AR Peters MC van Ryn J Gilmour SK. Thrombin inhibition and cisplatin block tumor progression in ovarian cancer by alleviating the immunosuppressive microenvironment. Oncotarget. (2016) 7(51):85291–305. 10.18632/oncotarget.13300 27852034 PMC5356737

[163] DeFeo K Hayes C Chernick M Ryn JV Gilmour SK. Use of dabigatran etexilate to reduce breast cancer progression. Cancer Biol Ther. (2010) 10(10):1001–8. 10.4161/cbt.10.10.13236 20798593

[164] Peraramelli S Zhou Q Zhou Q Wanko B Zhao L Nishimura T Thrombin cleavage of osteopontin initiates osteopontin’s tumor-promoting activity. J Thromb Haemost. (2022) 20(5):1256–70. 10.1111/jth.15663 35108449 PMC9289821

[165] Maqsood A Hisada Y Garratt KB Homeister J Mackman N. Rivaroxaban does not affect growth of human pancreatic tumors in mice. J Thromb Haemost. (2019) 17(12):2169–73. 10.1111/jth.14604 31393055 PMC6893077

[166] Smeda M Stojak M Przyborowski K Sternak M Suraj-Prazmowska J Kus K Direct thrombin inhibitor dabigatran compromises pulmonary endothelial integrity in a murine model of breast cancer metastasis to the lungs; the role of platelets and inflammation-associated haemostasis. Front Pharmacol. (2022) 13:834472. 10.3389/fphar.2022.834472 35295330 PMC8918823

[167] Shi K Damhofer H Daalhuisen J Ten Brink M Richel DJ Spek CA. Dabigatran potentiates gemcitabine-induced growth inhibition of pancreatic cancer in mice. Mol Med. (2017) 23:13–23. 10.2119/molmed.2016.00214 28182192 PMC5364111

[168] Kiricuta I Todorutiu C Muresian T Risca R. Prophylaxis of metastases formation by unspecific immunologic stimulation associated with heparintherapy. Cancer. (1973) 31(6):1392–6. 10.1002/1097-0142(197306)31:6<1392::AID-CNCR2820310614>3.0.CO;2-9 4709954

[169] Kenessey I Simon E Futosi K Bereczky B Kiss A Erdödi F Antimigratory and antimetastatic effect of heparin-derived 4–18 unit oligosaccharides in a preclinical human melanoma metastasis model. Thromb Haemost. (2009) 102(6):1265–73. 10.1160/TH09-01-0059 19967160

[170] Kim JY Alam F Chung SW Park J Jeon OC Kim SY Combinational chemoprevention effect of celecoxib and an oral antiangiogenic LHD4 on colorectal carcinogenesis in mice. Anticancer Drugs. (2014) 25(9):1061–71. 10.1097/CAD.0000000000000141 25003253

[171] Russo V Falco L Tessitore V Mauriello A Catapano D Napolitano N Anti-inflammatory and anticancer effects of anticoagulant therapy in patients with malignancy. Life (Basel). (2023) 13(9):1888. 10.3390/life13091888 37763292 PMC10532829

[172] Tagalakis V Blostein M Robinson-Cohen C Kahn SR. The effect of anticoagulants on cancer risk and survival: systematic review. Cancer Treat Rev. (2007) 33(4):358–68. 10.1016/j.ctrv.2007.02.004 17408861

[173] Wei Y Li W Lin J Wang D Dang C Diao D Prognostic value of anticoagulants in resectable gastric cancer. *PREPRINT* (Version 1) available at Research Square (2024). 10.21203/rs.3.rs-3982052/v1

[174] Sanford D Naidu A Alizadeh N Lazo-Langner A. The effect of low molecular weight heparin on survival in cancer patients: an updated systematic review and meta-analysis of randomized trials. J Thromb Haemost. (2014) 12(7):1076–85. 10.1111/jth.12595 24796727

[175] Montroy J Lalu MM Auer RC Grigor E Mazzarello S Carrier M The efficacy and safety of low molecular weight heparin administration to improve survival of cancer patients: a systematic review and meta-analysis. Thromb Haemost. (2020) 120(5):832–46. 10.1055/s-0040-1709712 32369854

[176] Goerner A. The influence of anticlotting agents on transplantation and growth of tumor tissue. J Lab Clin Med. (1931) 16(4):369–72.

[177] Läubli H Stevenson JL Varki A Varki NM Borsig L. L-selectin facilitation of metastasis involves temporal induction of *Fut7*-dependent ligands at sites of tumor cell arrest. Cancer Res. (2006) 66(3):1536–42. 10.1158/0008-5472.CAN-05-3121 16452210

[178] Cassinelli G Naggi A. Old and new applications of non-anticoagulant heparin. Int J Cardiol. (2016) 212(Suppl 1):S14–21. 10.1016/S0167-5273(16)12004-2 27264866

[179] Sakamoto J Yamashita Y Morimoto T Amano H Takase T Hiramori S Cancer-associated venous thromboembolism in the real World - From the COMMAND VTE registry. Circ J. (2019) 83(11):2271–81. 10.1253/circj.CJ-19-0515 31548438

[180] Haaland GS Falk RS Straume O Lorens JB. Association of warfarin use with lower overall cancer incidence among patients older than 50 years. JAMA Intern Med. (2017) 177(12):1774–80. 10.1001/jamainternmed.2017.5512 29114736 PMC5820735

[181] Agnelli G Becattini C Meyer G Muñoz A Huisman MV Connors JM Apixaban for the treatment of venous thromboembolism associated with cancer. N Engl J Med. (2020) 382(17):1599–607. 10.1056/NEJMoa1915103 32223112

[182] McBane RD 2nd Wysokinski WE Le-Rademacher JG Zemla T Ashrani A Tafur A Apixaban and dalteparin in active malignancy-associated venous thromboembolism: the ADAM VTE trial. J Thromb Haemost. (2020) 18(2):411–21. 10.1111/jth.14662 31630479

[183] Raskob GE van Es N Verhamme P Carrier M Di Nisio M Garcia D Edoxaban for the treatment of cancer-associated venous thromboembolism. N Engl J Med. (2018) 378(7):615–24. 10.1056/NEJMoa1711948 29231094

[184] Young AM Marshall A Thirlwall J Chapman O Lokare A Hill C Comparison of an oral factor Xa inhibitor with low molecular weight heparin in patients with cancer with venous thromboembolism: results of a randomized trial (SELECT-D). J Clin Oncol. (2018) 36(20):2017–23. 10.1200/JCO.2018.78.8034 29746227

[185] Lee CL Chen WS Wee Y Wang CS Chen WC Chiu TJ Direct oral anticoagulants are associated with superior survival outcomes than warfarin in patients with head and neck cancers. Cancers (Basel). (2022) 14(3):703. 10.3390/cancers14030703 35158969 PMC8833638

[186] Hiramoto K Akita N Nishioka J Suzuki K. Edoxaban, a factor Xa-specific direct oral anticoagulant, significantly suppresses tumor growth in colorectal cancer Colon26-inoculated BALB/c mice. TH Open. (2023) 7(1):e1–e13. 10.1055/s-0042-1758855 36751299 PMC9825203

